# Influence of pipe materials on in-building disinfection of *P. aeruginosa* and *A. baumannii* in simulated hot water plumbing

**DOI:** 10.1016/j.wroa.2023.100189

**Published:** 2023-06-15

**Authors:** Abraham Cullom, Mattheu Storme Spencer, Myra D. Williams, Joseph O. Falkinham, Amy Pruden, Marc A. Edwards

**Affiliations:** a Civil and Environmental Engineering, Virginia Tech, 1145 Perry St., 418 Durham Hall, Blacksburg, VA, 24061; b Department of Biological Sciences, Virginia Tech, Blacksburg, VA, 24061, USA

**Keywords:** Opportunistic pathogens, Drinking water, Disinfection, Premise plumbing, *Pseudomonas aeruginosa*, *Acinetobacter baumannii*

## Abstract

A framework is needed to account for interactive effects of plumbing materials and disinfectants on opportunistic pathogens (OPs) in building water systems. Here we evaluated free chlorine, monochloramine, chlorine dioxide, and copper-silver ionization (CSI) for controlling *Pseudomonas aeruginosa* and *Acinetobacter baumannii* as two representative OPs that colonize hot water plumbing, in tests using polyvinylchloride (PVC), copper-PVC, and iron-PVC convectively-mixed pipe reactors (CMPRs). Pipe materials vulnerable to corrosion (i.e., iron and copper) altered the pH, dissolved oxygen, and disinfectant levels in a manner that influenced growth trends of the two OPs and total bacteria. *P. aeruginosa* grew well in PVC CMPRs, poorly in iron-PVC CMPRs, and was best controlled by CSI disinfection, whereas *A. baumannii* showed the opposite trend for pipe material and was better controlled by chlorine and chlorine dioxide. Various scenarios were identified in which pipe material and disinfectant can interact to either hinder or accelerate growth of OPs, illustrating the difficulties of controlling OPs in portions of plumbing systems experiencing warm, stagnant water.

## Highlights

· We compared effects of four disinfectants in replicated premise plumbing systems.
· Copper pipes lost antimicrobial properties and iron pipes interfered with disinfection.
· Disinfectant efficacy for total bacteria did not predict reduction in inoculated pathogens.
· Copper silver ionization was effective for *P. aeruginosa* control.
· Chlorine and chlorine dioxide were sometimes effective for *A. baumannii*.

## Graphical abstract

**Figure:**
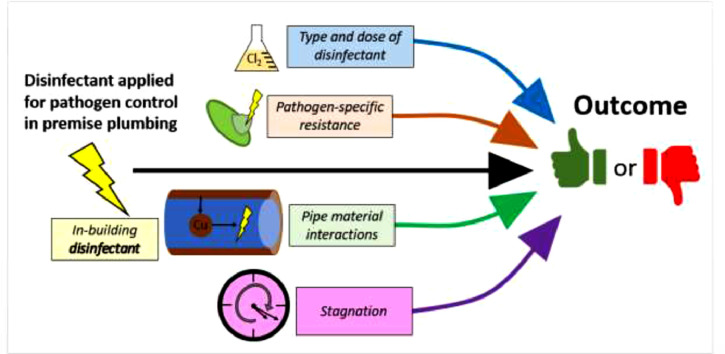

## 1 Introduction

Reliably controlling the growth of the opportunistic pathogens (OPs) encountered in premise (i.e., building) plumbing is especially important for nosocomial pathogens. In particular, *Pseudomonas aeruginosa* and *Acinetobacter baumannii* are found in hospital surfaces (Moultrie et al., 2011; Jefferies et al., 2012), drains, and wastewater collection systems (la Forgia et al., 2010; Breathnach et al., 2012). Both organisms also colonize potable water systems in hospitals and infect patients (Debast et al., 1996; Exner et al., 2005). Like most OPs, *P. aeruginosa* and *A. baumannii* are relatively resistant to secondary disinfectants compared to traditional fecal pathogens (Falkinham et al., 2015; Falkinham and Falkinham  III, 2015; Hayward et al., 2022) and include a variety of antibiotic resistant strains capable of causing deadly and untreatable infections (Centers for Disease Control and Prevention U.S., 2019). These bacteria are especially problematic in taps with mixing valves that temper hot water to a warm water range for scalding control (Wright, 2013). Recent research has demonstrated that, even with very hot water recirculating lines (> 55 °C), distal portions of plumbing such as risers are often in the warm temperature range (e.g., 32–42 °C) (Boppe et al., 2016) due to convective mixing (Rhoads et al., 2016). Such temperatures are unfortunately in the ideal growth range for most OPs (BROWN, 1957; Ohno et al., 2003), raising questions about effectiveness of strategies to control growth in such niches.

In-building disinfection is commonly employed for OP control in potable water plumbing, especially in facilities housing vulnerable populations, but recent research has brought to light the importance of considering how interactions with pipe materials can influence the efficacy of disinfection (Cullom et al., 2020; McCuin et al., 2022). In particular, iron, plastics, and copper are distinct in their interactions with disinfectants, which in turn will present advantages and disadvantages with respect to OP control (Falkinham et al., 2015; Cullom et al., 2020). For instance, plastic materials, such as PVC and PEX, are less reactive with chlorine disinfectant residuals than copper or iron (Zhang and Edwards, 2009), but may release organic carbon to the bulk water (Connell et al., 2016; Proctor et al., 2017), stimulating microbial growth (Proctor et al., 2017). Copper is often cited for its antimicrobial capacity, but it can encourage OP growth under certain circumstances for reasons that are not fully understood (Cullom et al., 2020). Iron and mild steel pipes corrode rapidly to form thick scales that can enhance OP growth by providing extensive surface area for biofilm attachment (Yang et al., 2012) and releasing nutrients (Morton et al., 2005). Ferric iron is extremely insoluble and is sometimes thought to limit growth of certain OPs under oxidizing conditions (Faraldo-Gómez and Sansom, 2003). Metallic pipes also are known to hasten the decay of chlorine or monochloramine (Al-Jasser, 2007; Zhang and Edwards, 2009; Ma et al., 2021). Further, copper-silver ionization (CSI) disinfection systems are expected to accelerate corrosion of iron (Cruse, 1971), which could render CSI disinfection less effective.

In-building disinfection in hospitals and other facilities is commonly employed to boost the same disinfectant added to the municipal water supply (typically chlorine or chloramine) or to add alternative disinfectants. However, there are many trade-offs to consider in the choice of secondary disinfectant. For example, chloramines are generally regarded as an effective control for *Legionella* and *P. aeruginosa* (Bédard et al., 2016; Gleason and Cohn, 2022), but can sometimes select for non-tuberculous mycobacteria (Waak et al., 2019; Pfaller et al., 2022). Free chlorine and chlorine dioxide are highly reactive, but, as a result, may decay quicker under stagnant conditions typical of premise plumbing, preventing penetration of biofilms where OPs reside (LeChevallier et al., 1990; Behnke and Camper, 2012). Highly reactive disinfectants are often at lower concentration or not detectable by the time the water reaches distal regions of the plumbing system (Nguyen et al., 2012).

In-building disinfectants can also potentially cause unintended public health concerns. As has been well documented for non-tuberculous mycobacteria, secondary disinfectants can sometimes kill competing background microbiota and inadvertently enrich OP populations (Bertelli et al., 2018). In addition to inducing shifts in the composition of the microbial community (Bertelli et al., 2018; Webster et al., 2021), there is growing evidence that disinfectants can differentially enrich OPs relative to other microbiota (Baron et al., 2014; Hayward et al., 2022), and also favor antibiotic resistant microorganisms (Sanganyado and Gwenzi, 2019). Such phenomena are likely exacerbated by the fact that there are numerous forces at play in premise plumbing causing disinfectants to decay below inhibitory concentrations in distal reaches, thereby creating niches for OP growth. Given that the wider microbial community can strongly influence OPs (Schwering et al., 2013), it is important to build an understanding of OP response to various disinfectants in the context of real-world systems colonized by natural microbiota, rather than extrapolation from observations of pure culture studies.

The purpose of this study was to conduct a head-to-head, replicated evaluation of the interactive effects of four in-building disinfectants (free chlorine, monochloramine, chlorine dioxide, and copper-silver ionization (CSI)) and three pipe materials (PVC, copper, and iron) for OP control in hot water plumbing under realistic conditions. Convectively-mixed pipe reactors (CMPRs) were employed for replicated comparison of experimental conditions (Fig S1). Through periodic decanting and refilling, these sealed pipe reactors simulate infrequently used, distal riser pipes from hot water recirculating lines which are known to generally be vulnerable to disinfectant decay and proliferation of OPs (Rhoads et al., 2016). *P. aeruginosa* and *A. baumannii* were inoculated and tracked in the CMPRs as two model OPs of concern, for which little information is available on suitable measures for in-building control. The synergistic and antagonistic effects of pipe-disinfectant combinations on water chemistry and microbiology were systematically evaluated to identify drivers of OP growth and provide holistic insights to their control.

## 2 Methods

### 2.1 Premise plumbing simulation using convectively mixed pipe reactors (CMPRs)

CMPRs are an inexpensive, space-efficient, and replicable means of simulating distal, stagnant outlets off of risers from hot water recirculation lines (Spencer et al., 2020). PVC, copper, and mild steel plumbing were simulated using sealed four-foot pipe segments composed of either ¾” PVC (Silver-line Plastics, Ashville, NC) (PVC CMPRs), half ¾” PVC and half type M copper pipe (McMaster-Carr, Elmhurst, IL) (copper-PVC CMPRs), or three-inches of ¾” mild steel pipe (McMaster-Carr, Elmhurst, IL) attached to forty-five-inches of ¾” PVC (iron-PVC CMPRs). All CMPRs were placed at a 60° angle with one end partially submerged in a 48 °C water bath and the other end exposed to ambient room temperatures, yielding an interior average water temperature of ∼37 °C and generating convective mixing currents (Fig S1). The ends of the CMPRs exposed to the ambient air were sealed with a silicone stopper and parafilm to provide access for water changes and to the bulk water and pipe for sampling. New pipes were seeded with backwash from *a* >2-year-old granular activated carbon filter. Pipes were aged for 10 weeks by decanting and refilling once weekly (weeks 1–2, to allow for seeded microbes to colonize) or twice weekly (weeks 3–10). See supporting information for influent water preparation (Section SI-1). CMPR effluent was treated by adding 7.5% sodium hypochlorite bleach (Clorox, Alpharetta, GA) to 10% v/v before disposal. During this period, chemical and biological water quality was profiled to verify the replicability of CMPRs as reported elsewhere (Spencer et al., 2020).

After pipe conditioning, water changes were increased to 3 times weekly to simulate an infrequent water use pattern. Each CMPR was inoculated with multidrug-resistant and antibiotic sensitive strains of both *P. aeruginosa* (ATCC 2795 and ATCC 2111, respectively) and *A. baumannii* (ATCC 2801 and ATCC 1789, respectively) at ∼1000/cells mL for each strain. After revival in rich media (LB), strains were acclimated to drinking water via sequential culturing in sterilized drinking water for a total 5 days, diluting the previous day's culture 1:10. Strains were inoculated into the CMPRs during all three water changes for one week, representing days 1, 3, and 5 of sequential culturing in drinking water. After inoculation, all water changes occurred in a Class II, Type A2 biosafety cabinet to maintain BSL-2 conditions. Thrice weekly water changes continued in the same manner for the remainder of the experiment.

Three phases of study were performed to evaluate effects of pipe material and the four residual disinfectants (Table 1). Six CMPRs (two triplicate sets) of each material were maintained as controls with no disinfectant added, whereas all other conditions were conducted in triplicate. In Phase 0, no disinfectants were added for 6 weeks to allow the inoculated OPs time to acclimate. All four strains were reinoculated immediately following sampling at the end of Phase 0 to ensure that they were present at similar levels before commencing disinfection in Phase 1. In Phase 1, very low doses of disinfectants, equal to 2.5% of the EPA National Primary Drinking Water Standard (NPDWS) for chlorine-based disinfectants (Agency, 2017) or secondary maximum contaminant level (MCL) of silver for CSI (U.S. Environmental Protection Agency and Agency, 2015) (Table 1) were added for 12 weeks to simulate the trace disinfectant levels that may result in infrequently used distal outlets of buildings employing in-building plumbing disinfection. Doses were increased in three increments in Phase 2, every 6 weeks over the next 18 total weeks, to achieve target influent levels of 6.25, 25 and 100% of the NPDWS (Cl_2_, NH_2_Cl, ClO_2_) or MCL (CSI). In Phase 3, water changes were continued for 7 weeks with no disinfectant residual to examine the potential for pathogen regrowth in a system where residual disinfectants are suddenly removed (e.g., the installation of a granular activated carbon filter or changes in water use patterns to longer stagnation). See Section SI-2 for disinfectant preparation details.

**Table 1:** Target Influent Disinfectant Doses (mg/L) for Phases 0–3.^a^.

	Phase 0	Phase 1		Phase 2			Phase 3
	No residual	Low Dose		Incrementally Increasing Doses			Regrowth
Disinfectant	Weeks −6–0	Weeks 1–6	Weeks 7 –12	Weeks 13–18	Weeks 19 –24	Weeks 25–30	Weeks 31–37
Cl_2_	0	0.1	0.1	0.25	1.0	4.0	0
NH_2_Cl	0	0.1	0.1	0.25	1.0	4.0	0
ClO_2_	0	0.02	0.02	0.05	0.2	0.8	0
CSI^b^	0	0.025	0.025	0.063	0.25	1.0^c^	0

a Biological samplings occurred in the final week of each indicated period.

b Dose expressed in terms of mg/L Cu, with a fixed ratio of Cu:Ag of 10:1 mg/L.

c Highest dose based on the secondary maximum contaminant level for silver.

### 2.2 Water chemistry sampling

Influent and effluent water chemistry parameters were measured every two weeks. After decanting effluent into 500 mL polypropylene bottles, pH, temperature (pH 110 meter with ATC probe, Oakton Research, Vernon Hills, Il.), and dissolved oxygen (DO) were measured (Thermo Scientific Orion 3-star meter). Aliquots were taken for total organic carbon (TOC) analysis using a Sievers Model 5300C autosampler according to Standard Method 5310 C. At the end of Phases 0, 1, and 2 (weeks −1, 11, and 29), total and soluble metals were measured in influent and effluent waters using ICP-MS (iCAP RQ ICP-MS; Thermo Fisher Scientific, Waltham, MA). Soluble metals were operationally defined as those passing through a 0.45-µm nylon filter (Whatman, Maidstone, UK).

Influent residual disinfectants were monitored with each water change in Phases 1 and 2. Effluent residuals of one disinfectant condition were monitored along with one set of the disinfectant-free pipes as a control on a rotating basis. Chlorine, monochloramine, and chlorine dioxide, as well as total and soluble copper and silver concentrations for CSI were measured using the same protocols described in Section SI-2.

### 2.3 Biological sampling and enumeration of A. baumannii, P. aeruginosa, and total bacteria

*A. baumannii* and *P. aeruginosa* were enumerated both by culture plate counts and quantitative polymerase chain reaction (qPCR) during one water change per week in weeks 0, 6, 12, 18, 24, 30, and 37. CMPR effluent was decanted into sterilized 500-mL polypropylene. Duplicate culture plates were used to quantify *P. aeruginosa* on Pseudomonas Isolation Agar (Sigma-Aldrich, St. Louis, MO) and *A. baumannii* on Leeds Acinetobacter Medium with Selective Supplement (Hardy Diagnostics, Santa Maria, CA). After spreading 100 µL of effluent onto the plate, plates were incubated for 24 h at 37 °C and colonies were counted. Influent water blanks and lab blanks (autoclave-sterilized DI water exposed to sampling conditions) were cultured each sampling.

The remaining effluent was filtered through 0.22-µm mixed cellulose-ester filters (Millipore, Billerica, MA) for DNA extraction. Additionally, at the end of Phases 1 and 2, biofilm swabs of the pipe surface (∼2 cm^2^) were collected. At the end of Phase 2, an additional biofilm swab was collected, with the attached particles resuspended in PBS via centrifugation before culturing via the same protocol as above. Filters and swabs were stored at −20 °C before further processing. DNA from filters and biofilm swabs was extracted using the FastDNA Spin Kit (MP Biomedicals, Solon, OH) according to the manufacturer's protocol. Influent water, lab, filter, and DNA extraction kit blanks were included in all downstream molecular analyses. qPCR was used to enumerate total bacteria employing universal primers targeting bacterial 16S rRNA gene (Suzuki et al., 2000). *P. aeruginosa* were enumerated by targeting the *oprL* gene (le Gall et al., 2013) and *A. baumannii* by targeting the 16S-23S rRNA intergenic spacer (ABITS) (Chang et al., 2005). Enumeration of the target pathogens via both culture-based and molecular methods allowed for the simultaneous evaluation of both monitoring approaches and investigation of conditions that induced viable‑but-not-culturable status. See Section SI-3 and Table S1 for additional details.

### 2.4 Data analysis

Data analysis was performed in R 3.6.1 using RStudio (R Core Team., 2020) (see Section SI-4 for details). Data were examined for normality using the Shapiro-Wilks test, as well as visual examination of probability density graphs and normal quantile plots, and most data sets (excepting that of temperature) were found to be non-normally distributed. A p-value of 0.05 was selected as a threshold for statistical significance. A non-parametric Kruskal-Wallis test followed by Dunn's test with Benjamini-Hochberg multiple comparisons correction was used to evaluate differences in biological and chemical water quality parameters. ANOVA and Tukey's Test were used for analysis of temperature data. Correlation analysis between OP levels and water quality parameters was performed using Spearman's rank correlation with a Benjamini-Hochberg multiple comparisons correction. We further explored the possible effects of the various measured water quality parameters on OPs in Phases 0–2 through correlation analysis and canonical correspondence analysis (CCA), pairing chemistry samplings closest to biological samplings. When correlating disinfectant doses to biological parameters, weeks 6 (intermediate low-dose timepoint) and 37 (regrowth timepoint) were eliminated to only include samplings that represented the endpoint of a dose level.

## 3 Results

### 3.1 Water chemistry is highly influenced by pipe material and disinfectant

**pH- pH** was highest over the duration of the experiment in iron-PVC CMPRs (7.93 ± 0.27; average ± SD), followed by copper-PVC (7.59 ± 0.22), and PVC (7.34 ± 0.25) (Fig S4). During Phase 2, application of disinfectants raised pH in certain circumstances. Compared to disinfectant-free pipes, effluent pH was higher in PVC CMPRs receiving chlorine (Dunn's Test, *p*<0.0001) and monochloramine (*p* = 0.038) in copper-PVC CMPRs receiving monochloramine (*p*<0.0001) and chlorine dioxide (*p* = 0.006), and in all four disinfectant conditions in iron-PVC CMPRs (*p*<0.05). During Phase 3, pH was higher in copper-PVC CMPRs that had received chlorine dioxide (Dunn's Test, *p* = 0.023) or CSI (*p* = 0.047).

**DO- DO** was strongly influenced by pipe type (Kruskal-Wallis, *p*<0.0001), with iron-PVC CMPRs notably deviating from the other CMPRs (Fig S5). When grouping the pipes based on disinfectant conditions in Phase 2, DO was lower in monochloramine PVC CMPRs than chlorine dioxide (average 0.52 mg/L lower), and lower in monochloramine iron-PVC CMPRs than both control and chlorine dioxide CMPRs (average 0.18 mg/L lower for both disinfectants). Use of monochloramine is often associated with low DO due to stimulation of aerobic nitrifying bacteria (Zhang, Griffin and Edwards, 2010). During Phase 3, DO was lower in PVC CMPRs receiving chlorine, monochloramine, and CSI than control CMPRs, as well as in copper-PVC CMPRs receiving chlorine and monochloramine (Dunn's Test, *p*<0.05).

**TOC- TOC** concentrations were highest in Phase 0, at 1.78–3.97 mg/L, perhaps due to high leaching potential from the new plastic pipes and seasonal peaks in the influent water derived from the public water supply. During Phases 1 and 2, low doses of chlorine and monochloramine increased the levels of final TOC by 0.2–0.3 mg/L in PVC and copper-PVC CMPRs (Fig S6). See Section SI-6 for further discussion of pH, DO, and TOC results.

**Fig. 1:**
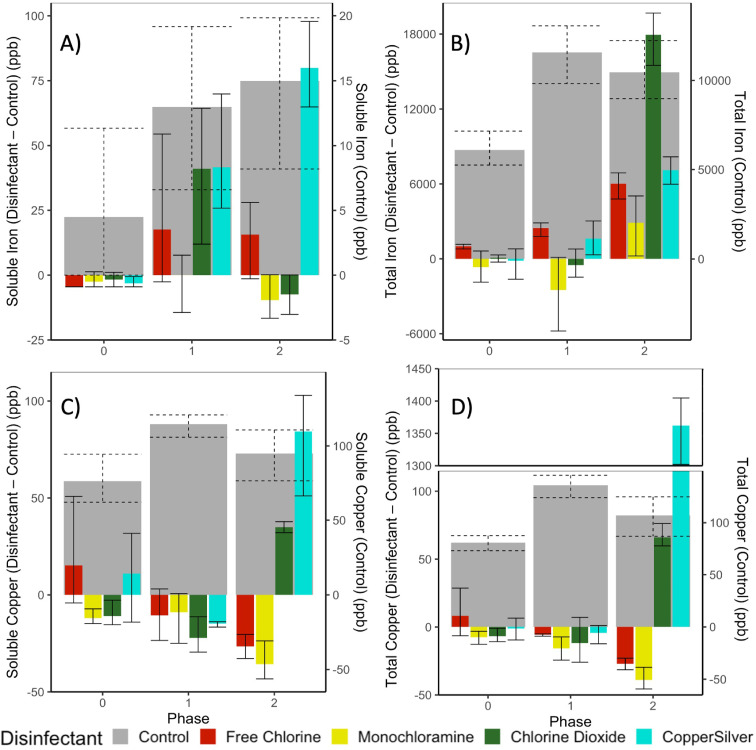
Differences in A) total and B) soluble iron concentrations measured in iron-PVC CMPR bulk water and C) total and D) soluble copper concentrations measured in copper-PVC within the various disinfectant conditions (Disinfectant) relative to the corresponding disinfectant-free conditions (control) at the end of Phase 0 (week −1), Phase 1 (week 11), and Phase 2 (week 29). Average concentrations in parts per billion (ppb) in the corresponding disinfectant-free condition (control) is plotted against the right axis. Error bars represent the nonparametric bootstrap 95% confidence interval of differences among measured concentrations in disinfectant conditions and the average concentration in the corresponding disinfectant-free condition, or the 95% confidence interval of measurements for the disinfectant-free conditions. Samples sizes are *n* = 3 for CMPRs that received disinfectant, *n* = 6 for controls.

**Metals**- Iron concentrations in iron-PVC CMPRs averaged 11.4 mg/L across the experiment (Fig 1B). Virtually all iron (>99%) was in particulate form (Fig 1A, 1B), as would be expected based on the high pH and low DO levels in the bulk water. Iron release was worsened by all disinfectants at their highest doses. Chlorine dioxide addition caused the most iron release (average 28.6 mg/L in Phase 2) and monochloramine the least. CSI addition also increased iron release by 67% at the highest doses. In contrast to iron, the release of copper to water was mostly soluble (typically ∼80–95%) (Fig 1C, 1D).

**Phosphate**- Corrosion inhibitor levels, as measured by soluble phosphorus, decreased by >40% when dosing CSI at the highest level for PVC and copper-PVC pipes (Fig S7), consistent with expectations that the addition of copper can precipitate phosphate (Song et al., 2021). In all iron-PVC CMPRs, the phosphate was in particulate form, likely due to formation of insoluble ferric phosphate compounds.

### 3.2 Stagnation and pipe-material interactions affect maintenance of disinfectant residual

**Fig. 2:**
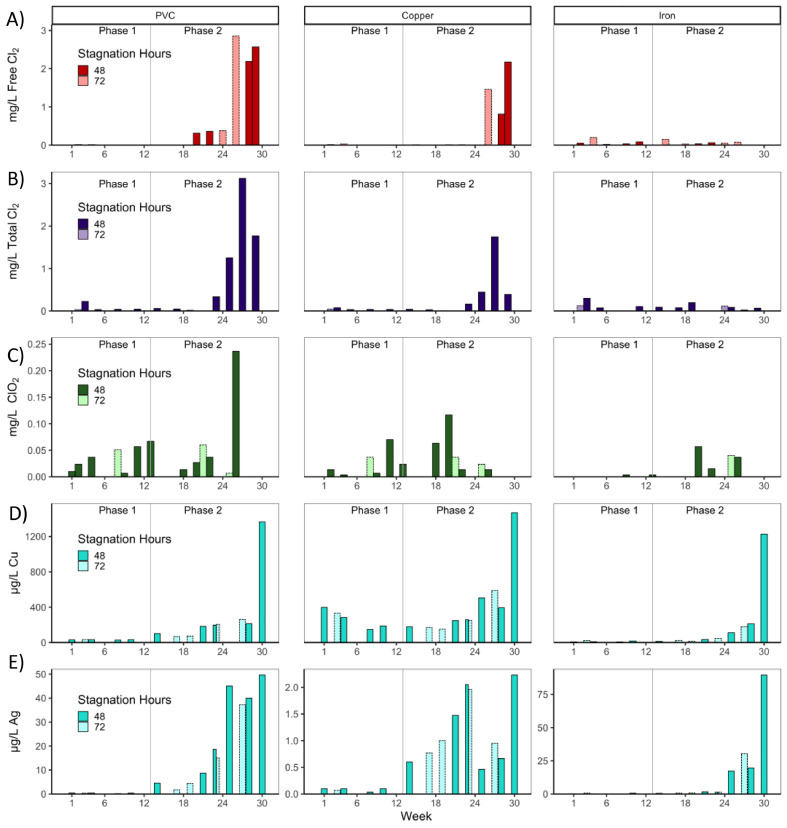
Remaining disinfectant residual following stagnation through Phase 1 (low disinfectant, weeks 1–12) and Phase 2 (increasing disinfectant, weeks 13–30) in A) free chlorine, B) monochloramine, C) chlorine dioxide, D) Cu in CSI disinfectant conditions, and E) Ag in CSI conditions. Mean effluent concentrations were measured prior to water change, after 48 (darker shade) or 72 (lighter shade with dashed borders) hours of stagnation.

At the highest disinfectant dose tested, none of the chlorine-based disinfectants were measurable in iron-PVC CMPR effluent after stagnation, whereas disinfectant was consistently detected in PVC CMPRs when dosed >1 mg/L (Fig 2). While tracking the rate of disinfectant decay in week 28 (Fig S8, Section SI-5), the half-life of chlorine and chlorine dioxide were 2–11 times longer in PVC pipe than in copper-PVC or iron-PVC pipe (Table S2). Monochloramine exhibited a half-life 16 times longer in PVC than in iron-PVC CMPRs, and the half-life in the copper condition was slightly longer than in the PVC condition.

Although copper and silver in CSI are conservative and cannot be degraded, they were removed from solution, possibly from a combination of sorption, deposition to the pipe wall, and precipitation from solution. At the highest CSI dose, the average copper concentrations were maintained above 0.4 mg/L in all CMPRs only in week 30, at which point copper concentrations in the effluent exceeded the influent levels for the first time, possibly because of flushing of previously deposited copper. Silver was nearly completely removed from copper-PVC CMPRs over the duration of the experiment, perhaps due to deposition corrosion. However, despite iron being a less noble metal than copper, silver remained detectable in iron-PVC CMPR effluents when dosed at high concentrations. This may be a result of complexation between silver and the iron present at high concentrations preventing deposition corrosion, as the soluble fraction of silver in copper-PVC CMPR effluent was roughly 7 times higher than that from iron-PVC CMPRs.

### 3.3 Influence of pipe materials on total bacteria, P. aeruginosa, and A. baumannii levels

#### 3.3.1 Response of bacterial targets to pipe material in bulk water

**Fig. 3:**
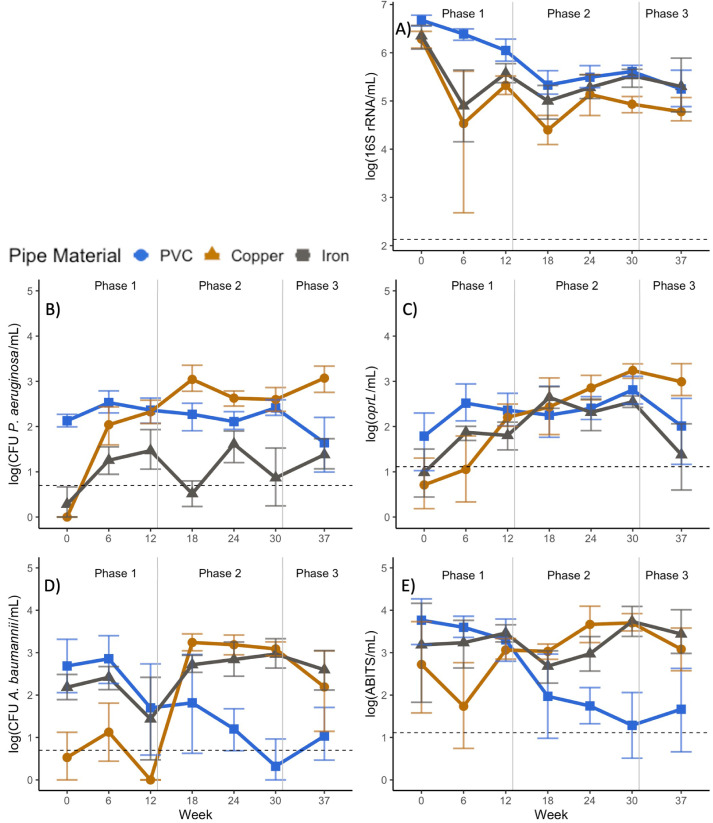
Effects of pipe materials on bacterial targets in absence of disinfectant. Levels of A) total bacterial 16S rRNA gene copies, B) *P. aeruginosa* culture counts and C) *P. aeruginosa oprL* gene copies, D) *A. baumannii* culture counts, and E) *A. baumannii* 16S-23S intergenic spacer copies in disinfectant-free (control) CMPRs over the duration of the experiment. Error bars represent 95% non-parametric bootstrap confidence intervals.

Trends in the disinfectant-free conditions were evaluated to assess the background response of the target microbial populations to the pipe materials. CMPRs were found to be capable of maintaining high levels of *P. aeruginosa, A. baumannii*, and total bacteria (Table S3, Fig 3), which confirmed the influence of the biofilm on the bulk water microbial communities and enabled evaluation of the effect of pipe materials.

**Total bacteria**- During Phases 0 and 1, total bacterial gene copy numbers among disinfectant-free CMPRs varied by pipe material (Kruskal-Wallis, *p*<0.0001) and were highest in the PVC followed by iron-PVC conditions. These levels were on average 0.99 and 0.76 logs higher in the bulk water of PVC CMPRs than copper-PVC or iron-PVC CMPRs during Phases 0 and 1, and 0.65 and 0.21 logs higher during Phase 2.

***P. aeruginosa* - *P. aeruginosa*** CFUs also varied as a function of pipe material (Kruskal-Wallis, *p*<0.0001) (Fig 3B, 3C) and were highest in PVC CMPRs during Phase 0, remaining relatively constant thereafter. In the iron-PVC and copper-PVC CMPRs, *P. aeruginosa* CFUs were below the detection limit during Phase 0, but increased in Phases 2 and 3 such that the final trend was copper-PVC > PVC > iron-PVC. This may be associated with the observed correlation between *P. aeruginosa* gene copies and DO (Section SI-7), which tended to be lower in iron-PVC CMPRs.

Numbers of *P. aeruginosa* gene copies indicated a similar, but more modest, effect of pipe material (Kruskal-Wallis, *p*<0.0001), indicative of the significant but modest correlations between culture and molecular methods (Section SI-9). As with CFUs, *P. aeruginosa* gene copies began low in copper-PVC relative to the other materials, but increased over time. Relative abundance (i.e., *P. aeruginosa* gene copies/16S rRNA gene copies) also tended to be 0.5–2 logs higher in copper-PVC than in PVC CMPRs (Dunn's Test *p* = 0.006) and iron-PVC pipes (*p* = 0.004).

***A. baumannii*- *A. baumannii*** CFUs also indicated distinct responses to the pipe materials in the disinfectant-free conditions (Kruskal-Wallis, *p*<0.0001). At week 0, culturable levels were higher in the PVC CMPRs than copper-PVC or iron-PVC by 2.16 and 0.50 logs, but fell below detection limits by the end of Phase 2 (Fig 3D, 3E). In contrast to *P. aeruginosa, A. baumannii* levels tended to remain stable (within ∼1-log) in iron-PVC CMPRs. *A. baumannii* CFU in copper-PVC CMPRs increased ∼2.5 logs over the course of the experiment. As was observed for CFUs, *A. baumannii* gene copies also steadily decreased in PVC CMPRs but remained detectable. The relative abundance was generally higher in copper-PVC CMPRs than other pipe types.

#### 3.3.2 Influence of pipe materials on total bacteria, P. aeruginosa, and A. baumannii in biofilm

Total bacteria- In the disinfectant-free CMPRs sampled for biofilm at week 12, total bacterial 16S rRNA gene levels were lower in copper-PVC than PVC or iron-PVC conditions (Fig S9). In contrast to bulk water trends, levels in PVC biofilms were not significantly different from levels in iron-PVC biofilms (Dunn's Test, *p*>0.05). A global decrease was observed in all bacterial targets in biofilm between weeks 12 and 30, possibly because of seasonal changes of the TOC of the influent water. By week 30, no significant differences were observed in terms of total bacterial gene copies measured in biofilm as a function of pipe material (Dunn's Test, *p*>0.05), mirroring the convergence towards levels observed in the bulk water of copper-PVC CMPRs.

*P. aeruginosa* and *A. baumannii* measured in biofilm via both CFU- and qPCR-based enumeration did not exhibit significant differences based on pipe material during either the week 12 or week 30 timepoint (Dunn's Test, *p*>0.05).

### 3.4 Identifying threshold disinfectant concentrations for OP control with different pipe materials

#### 3.4.1 Response of pathogens to disinfectants in bulk water

**Fig. 4:**
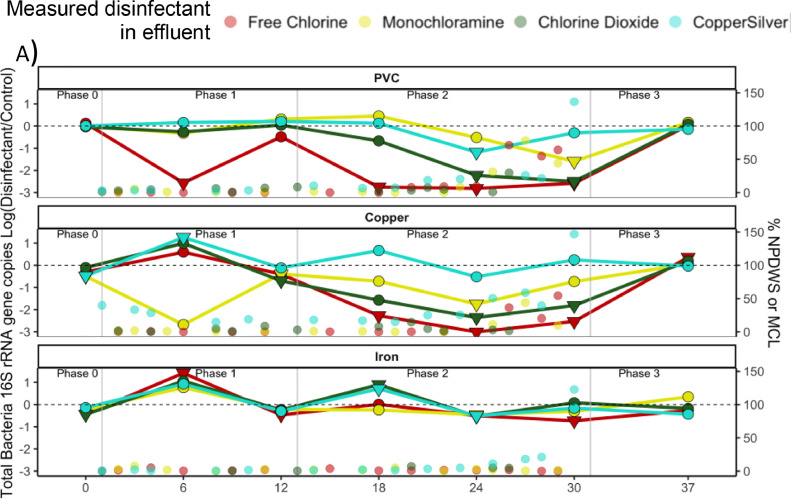

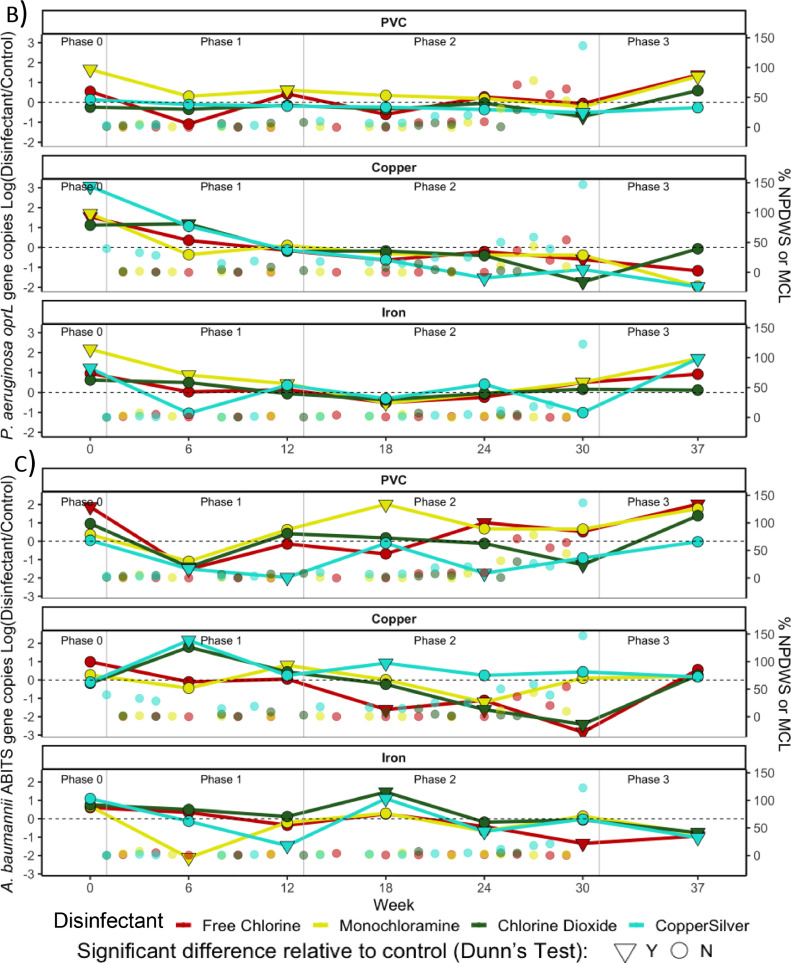
Ratio of A) total bacteria (16S rRNA gene copy numbers), B) *P. aeruginosa* (*oprL* gene copy numbers), and C) *A. baumannii* (16S-23S rRNA gene intergenic spacer (ABITS) copy numbers) measured by qPCR in disinfectant versus disinfectant-free conditions in CMPR bulk water. Symbols indicate statistical significance relative to disinfectant-free control measurements via Dunn's Test (● = *p>*0.05, ▼ = *p<*0.05) for each sampling timepoint. Mean remaining disinfectant residual following stagnation as a percentage of the NPDWS or MCL are plotted against the y axis for each condition. Samples sizes are *n* = 3 for CMPRs that received disinfectant, *n* = 6 for disinfectant-free controls.

Total bacteria- In Phase 1, total bacterial 16S rRNA genes were reduced relative to disinfectant-free CMPRs by low doses of chlorine (0.1 mg/L) in PVC CMPRs and low doses of chlorine dioxide (0.025 mg/L) in copper-PVC (Fig 4A, S10A). As doses were increased during Phase 2, chlorine reduced levels of 16S rRNA genes by an average of 2.72 logs in PVC and 2.60 logs in copper-PVC CMPRs. Similarly, chlorine dioxide reduced levels of 16S rRNA genes by 1.80 logs in PVC and by 1.92 logs in copper-PVC CMPRs. In iron-PVC CMPRs, chlorine was the only disinfectant to reduce total bacteria numbers, but only modestly (<1-log) and at the highest dose. Monochloramine and CSI exhibited more modest reductions in the PVC and copper-PVC CMPRs. Monochloramine reduced levels of bacterial 16S rRNA >1 log only in PVC CMPRs during week 30 (4 mg/L) and copper-PVC CMPRs during week 24 (1 mg/L). CSI only did so in the copper-PVC CMPRs, during week 24 (0.25:0.025 mg/L Cu:Ag).

*P. aeruginosa-* By week 12 of Phase I, CSI reduced *P. aeruginosa* CFUs to levels below those of the control and other disinfectants (0.025:0.0025 mg/L Cu:Ag) in all pipe materials (Dunn's Test, *p*<0.05) (Fig S11A, S12A). After week 12, all disinfectants reduced culturable *P. aeruginosa* below the limit of detection for the remainder of Phase 2 (Fig S11A). CSI was the only disinfectant correlated with reduction in culturable levels, in both PVC (Spearman's rho=−0.79, *p* = 0.0004) and copper-PVC CMPRs (Spearman's rho=−0.70, *p* = 0.004), and the only disinfectant that appeared effective for culturable *P. aeruginosa* across all pipe types.

Chlorine also effectively reduced culturable *P. aeruginosa* in the non-iron CMPR conditions. Within copper-PVC CMPRs, culturable *P. aeruginosa* was lower in chlorine conditions at two timepoints, one during Phase 1 (weeks 12) and one during lower dosing in Phase 2 (week 18). The highest doses of chlorine (4 mg/L) also reduced *P. aeruginosa* CFUs and gene copy levels in copper-PVC CMPRs and CFUs in PVC CMPRs (Dunn's Test). High doses of chlorine dioxide also effectively reduced *P. aeruginosa* in PVC CMPRs.

While qPCR still indicated that CSI was the most effective disinfectant for *P. aeruginosa*, reductions were small and only significant in copper-PVC CMPRs in the final two timepoints of Phase 2 (0.8:0.08, 1:0.1 mg/L Cu:Ag), with levels 1.54 and 1.1 logs lower than the controls (Dunn's Test, *p* = 0.015 and 0.021, respectively) and only 0.54 logs lower in PVC CMPRs during the final timepoint (*p*>0.05) (Fig 4B).

Monochloramine did not measurably reduce *P. aeruginosa* gene copies at any timepoint, while chlorine only did so at the highest dose (4 mg/L) in copper-PVC CMPRs. Chlorine dioxide also only successfully reduced *P. aeruginosa* gene copy numbers at the highest dose (0.8 mg/L), in PVC and copper-PVC pipes. Chlorine increased the relative abundance of *P. aeruginosa* the most in contexts where it appeared most effective for total bacteria: at higher doses in PVC CMPRs (Fig S13A). In fact, most disinfectant-pipe-material combinations were associated with an increase in the relative abundance of *P. aeruginosa* by week 30.

*A. baumannii* - In contrast to the efficacy of CSI observed for *P. aeruginosa* control, CSI did not consistently reduce culturable *A. baumannii* (Fig S11B, S12B). The most effective disinfectants for *A. baumannii* control across the CMPR conditions were chlorine and then chlorine dioxide in copper-PVC CMPRs, which reduced culturable numbers 3.09 and 2.02 logs, respectively, at the corresponding highest doses.

Chlorine and chlorine dioxide reduced *A. baumannii* gene copies by 2.84 and 2.42 logs in copper-PVC CMPRs at the highest dose (Fig 4C). In contrast to culturable results, CSI reduced *A. baumannii* gene copies by 1.37 and 0.92 logs in weeks 24 and 30 in PVC CMPRs. Monochloramine did not have a measurable effect on *A. baumannii* levels in bulk water.

#### 3.4.2 Response of pathogens to disinfectant in biofilm

**Fig. 5:**
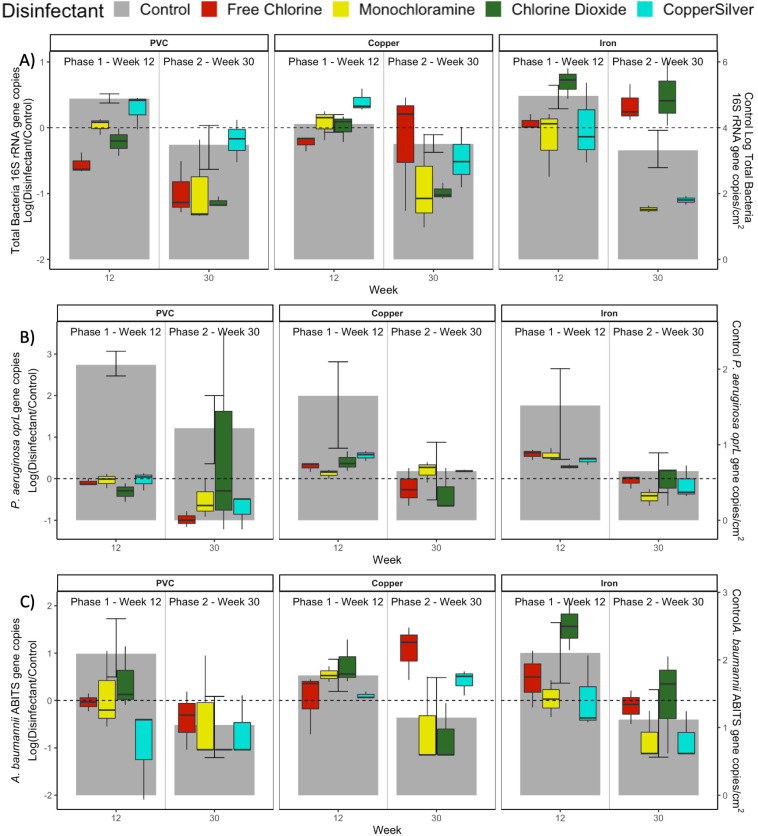
Differences in the biofilm numbers of A) total bacterial 16S rRNA gene copies, B) *P. aeruginosa oprL* gene copies, and C) *A. baumannii* 16S-23S rRNA gene intergenic spacer (ABITS) copies between disinfectant and disinfectant-free conditions in the CMPRs at the end of Phase 1 and Phase 2. Average log-transformed levels per cm^2^ in disinfectant-free conditions are displayed in gray, with error bars representing 95% non-parametric bootstrap confidence intervals. Average log-transformed levels per cm^2^ in disinfectant-free conditions are displayed in gray, with error bars representing 95% non-parametric bootstrap confidence intervals. Samples sizes are *n* = 3 for CMPRs that received disinfectant, *n* = 6 for controls.

Total Bacteria- Perhaps owing to the general reduction in bacterial levels in week 30, very few statistical differences were observed for any parameter. As observed for the bulk water, chlorine-based disinfectants reduced total bacterial levels in biofilm ∼1-log in PVC CMPRs, and both monochloramine and chlorine dioxide did so in copper-PVC CMPRs (Fig 5A, S15A). CSI was again the least effective for reducing total bacterial 16S rRNA genes in biofilm for both of these pipe materials. Monochloramine and CSI were the only disinfectants to reduce levels of 16S rRNA genes in the biofilm of iron-PVC CMPRs when measured at week 30 (1.24 and 1.10 logs lower than the controls, respectively via Dunn's Test).

*P. aeruginosa* and *A. baumannii* – In biofilm, neither *P. aeruginosa* nor *A. baumannii* culture or gene copy numbers were reduced by disinfectant (Dunn's Test, *p*>0.05, Fig 5B, 5C, S16, S17). This was in stark contrast to the effectiveness of disinfectants observed in the bulk water. The observable, not statistically significant trends in culturable levels of the two OPs behaved similarly to one another within the biofilm, which was not the case in the bulk water. For example, CSI reduced culturable levels relative to the control the most within biofilm for both OPs and all pipe types.

### 3.5 Recovery of pathogens following removal of disinfectants

After removing disinfectant in Phase 3, total bacteria and both OPs exhibited near full regrowth to pre-disinfectant levels within 7 weeks in most conditions. One notable exception were the CMPRs that were previously dosed with CSI, where culturable *P. aeruginosa* remained below the detection limit in Phase 3 in all corresponding PVC and copper-PVC CMPRs but one. All other previously disinfected PVC and copper-PVC CMPRs (except copper-PVC CMPRs that had previously been dosed with chlorine) exhibited regrowth of culturable *P. aeruginosa* to levels that were comparable to the controls (Dunn's Test, *p*>0.05). CSI also appeared to demonstrate a continued reduction in culturable *A. baumannii* in copper-PVC CMPRs (0.89 logs lower than the control). However, in all other PVC and copper-PVC CMPRs, *A. baumannii* regrew when disinfectant was removed. In fact, in the conditions that had previously been disinfected with chlorine, monochloramine, and chlorine dioxide in Phase 2, *A. baumannii* regrew to levels that were even higher than the corresponding disinfectant-free controls.

In terms of gene copies, *P. aeruginosa* were 0.26 and 1.97 logs lower in CMPRs that had previously been disinfected with CSI than in the corresponding PVC and copper-PVC control CMPRs, but this difference was not statistically different. In the iron-PVC CMPRs, levels of *P. aeruginosa* were higher in the CMPRs that had been dosed with CSI than in the controls (Dunn's Test, *p* = 0.04). CSI did not exhibit a continued reduction in *A. baumannii* gene copies. Concerningly, both culture-based and molecular results indicated that *A. baumannii* increased to higher levels in PVC CMPRs that received any chlorine-based disinfectant than in the controls that were never disinfected. In most cases, the relative abundance (i.e., normalized to total bacterial 16S rRNA genes) of the OPs decreased after removal of disinfectants (Fig S13, S14), particularly in CMPRs that had received chlorine and for *P. aeruginosa* gene copies. However, the relative abundance of *P. aeruginosa* increased >2-logs after the termination of CSI dosing in iron-PVC CMPRs.

## 4 Discussion

### 4.1 Overarching effects of pipe materials on water chemistry and ability to control OPs

Pipe materials and disinfectants had pronounced effects on the water chemistry of the CMPRs, which in turn influenced OP growth potential. Iron, and even copper, released from pipes has been suspected to sometimes provide limiting nutrients for OPs (Samanovic et al., 2012). Increased iron has previously been associated with increased growth of L. *pneumophila* (Stout et al., 1992). Iron pipe released iron at levels 38 times the EPA national secondary drinking water standard of 0.3 mg/L (U.S. Environmental Protection Agency and Agency, 2015) and reduced phosphate levels, even though only ∼6% of the CMPR length was composed of iron. The observed associations between released iron and OPs emphasize the importance of corrosion control (Rhoads et al., 2017) and flow regimes in premise plumbing.

Copper is largely considered as an antimicrobial, but it is also a trace nutrient and correspondingly can sometimes increase, decrease, or have no effect on certain OPs (e.g., L. *pneumophila* and *A. baumannii*) (Cullom et al., 2020). Iron and copper released to solution also are problematic because of the demand that they impose on chlorine-based disinfectants (Al-Jasser, 2007; Zhang and Edwards, 2009; Rhoads et al., 2017). In this study, rapid loss of all chlorine-based disinfectants was observed in the iron-PVC CMPRs. Further, disinfectants increased iron corrosion, creating a vicious cycle in which interactions between iron pipe and disinfectant made it impossible to maintain a residual, while also increasing iron nutrient concentration.

In the CSI condition, observed elevated iron aligned with expectations of copper-induced iron release due to copper-iron deposition corrosion (Cruse, 1971). This increased corrosion may have generated more surface area for biofilm growth or bioavailable iron, which may partially explain why *P. aeruginosa* gene copy levels in iron-CMPRs regrew to the highest level in those which received CSI after cessation of disinfection. A more detailed analysis of corrosion in the CMPRs and comparison to other studies is provided in Section SI 8.

The effects of pipe materials on water quality and the correspondingly high bacterial levels observed in this study underscore the important role of in-building disinfectants, especially in buildings with vulnerable populations. However, this study also elucidated the challenges associated with maintaining effective disinfectant levels in the stagnant, distal reaches of hot water plumbing, particularly for chlorine-based disinfectants. Warm and hot water plumbing are often associated with higher bacterial growth (Salehi et al., 2018; Pierre et al., 2019), organics leaching from plastic (Salehi et al., 2020), and metallic corrosion (Salehi et al., 2020), all of which are barriers to disinfectant delivery compared to cold water. However, stagnant conditions confound disinfectant maintenance even in cold water (Nguyen et al., 2012; McCuin et al., 2022). In this study, disinfectants almost never persisted above the detection limit in iron-PVC CMPRs. Even in PVC and copper-PVC CMPRs, chlorine-based disinfectant residuals were not consistently maintained over common state minimum levels for free and total chlorine (0.2 mg/L and 0.5–1.0 mg/L, respectively (Pennsylvania Department of Environmental Protection., 2015)) in distribution systems until dosed at the highest levels.

### 4.2 Effect of pipe materials in absence of disinfectants

Pipe materials influenced OP levels in absence of disinfectants. PVC CMPRs initially contained the highest levels of both OPs, by both culture- and molecular-based measurements, but levels of gene copy numbers in copper-PVC CMPRs overtook those in PVC and iron-PVC CMPRs by the end of the study. This is particularly interesting given the default assumption that copper pipe exhibits antimicrobial properties (Cullom et al., 2020) and is recommended as the pipe material of choice for OP control (Cervantes et al., 2013). A recent review noted that copper's effects on microbial growth control are actually variable and dependent on water quality characteristics (Cullom et al., 2020). The present study demonstrates that copper's antimicrobial effects are subject to change over time and can vary with water quality, water use patterns, and the pipe material itself. Thus, copper should not be regarded as ‘silver bullet’ for in-building OPs control.

### 4.3 Selecting an optimal disinfectant for in-building disinfection

CMPRs enabled a high-fidelity simulation of four disinfection regimes, demonstrating the limits of chemical disinfection for OPs control in stagnant, hot-water plumbing. Total bacteria were reduced to a greater extent by all four disinfectants when compared to the two OPs, aligning with the understanding that OPs tend to be relatively disinfectant resistant (Karumathil et al., 2014; Falkinham and Falkinham  III, 2015; Falkinham et al., 2015). These findings add further evidence of the inadequacy of general microbial targets employed in drinking water quality monitoring for informing the status of OPs, as well as the limitations of culture methods for detecting OPs in low levels or a viable‑but-not-culturable state.

The threshold of disinfectant required to reduce OP growth was found to be both highly material- and organism-specific. While *P. aeruginosa* and *A. baumannii* generally required greater doses of a disinfectant to demonstrate reductions than total bacteria, optimal disinfection strategies for the two organisms were not well aligned. Chlorine dioxide was the most effective oxidizing disinfectant for both organisms, in agreement with previous research (Hinenoya et al., 2015), while monochloramine was the least effective, contrasting previous bench-scale studies (Bédard et al., 2016). Interestingly, the application of high concentrations of chlorine dioxide within copper-PVC CMPRs and subsequent release of copper appeared to leverage both chlorine dioxide's capacity to control total bacteria and *A. baumannii* as well as copper's ability to inactivate *P. aeruginosa*, a unique synergy between pipe material and disinfectant.

CSI proved to be the most effective at suppressing *P. aeruginosa* levels, which is consistent with previous research noting that *P. aeruginosa* appears more susceptible to copper-inactivation than *A. baumannii* (Cullom et al., 2020). CSI also exhibited a sustained effect for *P. aeruginosa* control, with culturable levels remaining below the detection limit in most pipes in Phase 3, possibly because copper and silver persisted with the biofilm after dosing. There was some evidence of efficacy of CSI against *A. baumannii*; however, this was only confirmed using culture-based methods and it is possible that viable‑but-not-culturable forms were induced.

Notably, application of any of the chlorine-based disinfectants in PVC CMPRs was associated with an increase in *A. baumannii* numbers in Phase 3. Since PVC sustained disinfectant levels the longest, it is possible that, in these conditions, stronger shifts in the general microbial community were incurred, and *A. baumannii* was subsequently able to better regrow in the system once disinfectants were removed.

### 4.4 Biofilms were not effectively disinfected

No pipe-based differences or disinfectant-based reductions compared to the control were observed within the biofilm, where OPs tend to reside. The variable response of OPs in bulk water versus biofilm is noteworthy. Since OPs tend to be biofilm-associated, OPs control and OPs monitoring sometimes target the biofilm phase. However, this study indicates that in certain settings, targeting the biofilm for disinfection may prove less practical than inactivating OPs that enter the bulk water phase. Additionally, this work suggests that biofilm monitoring may not accurately identify risk factors associated with growth in the bulk water nor reflect risks to consumers.

### 4.5 Trends in responses of different microbial targets to the pipe and disinfectant conditions

Trends in total bacterial 16S rRNA genes provided insight into the effects of the CMPR conditions on bacterial microbiota in general. Total bacteria levels were initially highest in PVC CMPRs, likely because of high levels of leaching of organic carbon from the new pipes, but decreased over time, consistent with decreased TOC release from PVC pipes with time (Connell et al., 2016). *P. aeruginosa* levels were also initially highest in PVC CMPRs and lowest in iron-PVC CMPRs over the course of the experiment, in contrast to *A. baumannii*, which was sustained in the iron condition. This pattern is consistent with prior studies that reported more *P. aeruginosa* growth on plastic pipes than copper ones (Cervantes et al., 2013) and lower *A. baumannii* growth than *P. aeruginosa* growth on PVC (Wang et al., 2012). However, the findings contrast with others that have shown higher growth in conditions with iron pipes than PVC pipes (Wang et al., 2012).

Relative abundance is of interest from an ecological perspective, as it provides an indication of relative efficacy of the disinfectants as compared to total bacteria. Remarkably, all disinfectants tended to increase relative abundance of OPs. This was especially true in cases where a disinfectant did not appear effective for the OP, e.g. chlorine and *P. aeruginosa*. The most dramatic increase in relative abundance occurred in iron-PVC CMPRs that had received CSI between weeks 30 and 37. This result may be a combination of several factors. CSI's specificity for *P. aeruginosa* inactivation, complexation of influent copper with corroded iron in the bulk water preventing copper from remaining within the biofilm or depositing onto the pipe surface, or damage to the microbial community, which allowed *P. aeruginosa* to further colonize the pipe environment may have all contributed to the regrowth.

*A. baumannii* levels were more strongly correlated with total bacteria than levels of *P. aeruginosa*. In certain contexts, *A. baumannii* was better controlled by disinfectants that also proved effective for total bacteria, implying that *A. baumannii* aligns better with traditional water quality monitoring parameters than *P. aeruginosa*. Previous research has also indicated that OPs inconsistently correlate with total bacteria or HPCs (Williams et al., 2015). *Pseudomonas* spp. have been identified as early colonizers within drinking water distribution systems (Douterelo et al., 2014). Thus, their niche within premise plumbing may be to colonize where other bacteria are less abundant.

## 5 Conclusions

This study provides a comprehensive, side-by-side comparison of the efficacy of four in-building disinfectants for the control of two distinct OPs under realistic premise plumbing conditions representative of three common pipe materials. Interactive effects of the disinfectants with the pipe materials were found to create unique microenvironments that can undermine control of *P. aeruginosa* or *A. baumannii*. The findings help to better inform selection of in-building disinfectant types and doses for a given pipe material or, otherwise, if certain pipe materials (e.g., iron) are present, then no disinfection approach may realistically be effective. Overall, the results here highlight the dependency of disinfection effectiveness on plumbing material, as well as limitations of the chemical-disinfection paradigm for OPs control under typical premise plumbing conditions. The following points summarize key observations gained from this study:

• In the unfavorable scenario for premise plumbing pathogen control that the CMPRs simulated, total bacteria, *P. aeruginosa*, and *A. baumannii* responded distinctly to pipe material and disinfectant dose. None appeared inhibited by copper piping by the end of the experiment.
• Total bacteria were a poor proxy for control of either pathogen and no disinfectant reduced levels for either OP in all plumbing materials. Disinfectants typically only had an impact on OPs at doses 25–100% of the NPDWS or MCL, which is difficult to deliver to distal sites.
• It is concerning that higher doses of disinfectant frequently increased the relative abundance of these two OPs, especially of *P. aeruginosa*. Removal of disinfectant typically led to total regrowth of both OPs within 7 weeks. In some circumstances, OPs regrew to levels greater than those in pipes that had never received disinfectant.
• CSI proved to be a targeted means *P. aeruginosa* control, with a sustained effect even after dosing cessation. There was some evidence of efficacy of CSI against *A. baumannii*, but this was not consistent across all pipe materials.

## CRediT authorship contribution statement

**Abraham Cullom:** Methodology, Formal analysis, Writing – original draft, Writing – review & editing, Visualization. **Mattheu Storme Spencer:** Conceptualization, Methodology, Formal analysis, Investigation, Writing – review & editing, Funding acquisition. **Myra D. Williams:** Methodology, Investigation. **Joseph O. Falkinham:** Conceptualization, Methodology, Writing – review & editing. **Amy Pruden:** Conceptualization, Methodology, Writing – review & editing, Project administration, Funding acquisition. **Marc A. Edwards:** Conceptualization, Methodology, Writing – review & editing, Project administration, Funding acquisition.

## Declaration of Competing Interest

The authors declare the following financial interests/personal relationships which may be considered as potential competing interests

Abraham Cullom reports financial support was provided by Center for Disease Control and Prevention. Abraham Cullom reports financial support was provided by NSF Graduate Research Fellowship Program.

## Data Availability

Data will be made available on request.

## Appendix Supplementary materials

Image, application 1
