# Supplementary material for: Influence of pipe materials on in-building disinfection of P. aeruginosa and A. baumannii in simulated hot water plumbing
Source: Water Res X. 2023 Jun 15;21:100189. doi: 10.1016/j.wroa.2023.100189 (PMC10719577; doi:10.1016/j.wroa.2023.100189)
Supplement: Supplementary file 1 [file mmc1.docx]

**Supporting Information**

**Influence of pipe materials on in-building disinfection of *P. aeruginosa* and *A. baumannii* in simulated hot water plumbing**

Abraham Cullom^1^, Mattheu Storme Spencer^1^, Myra D. Williams^2^, Joseph O. Falkinham III^2^, Amy Pruden^1^, Marc A. Edwards^1^

^1^ Civil and Environmental Engineering, Virginia Tech, 1145 Perry St., 418 Durham Hall, Blacksburg, VA 24061

^2^ Department of Biological Sciences, Virginia Tech, Blacksburg, VA 24061

**Contents**

[Section SI-1. Influent water preparation 3](#_Toc124777173)

[Section SI-2. Disinfectant preparation 3](#_Toc124777174)

[Section SI-3. qPCR methods and quality control 3](#_Toc124777175)

[Section SI-4. Software packages used in data analysis 3](#_Toc124777176)

[Section SI-5. Disinfectant decay and half-life calculation 3](#_Toc124777177)

[Section SI-6. Discussion of water chemistry findings 4](#_Toc124777178)

[Section SI-7. OPs correlation with water quality parameters 4](#_Toc124777179)

[Section SI-8. Comparison of pipe corrosion results to previous studies 5](#_Toc124777180)

[Section SI-9. Comparison of culture and qPCR methods 5](#_Toc124777181)

[Section SI-10. Disinfectant efficacy compared to previous studies 6](#_Toc124777182)

[Section SI-11. Analysis of temperature data 6](#_Toc124777183)

[SI Tables 7](#_Toc124777184)

[Table S1 7](#_Toc124777185)

[Table S2 7](#_Toc124777186)

[Table S3 9](#_Toc124777187)

[Table S4 10](#_Toc124777188)

[SI Figures 11](#_Toc124777189)

[Figure S1 11](#_Toc124777190)

[Figure S2 12](#_Toc124777191)

[Figure S3 13](#_Toc124777192)

[Figure S4 14](#_Toc124777193)

[Figure S5 15](#_Toc124777194)

[Figure S6 16](#_Toc124777195)

[Figure S7 17](#_Toc124777196)

[Figure S8 18](#_Toc124777197)

[Figure S9 19](#_Toc124777198)

[Figure S10 21](#_Toc124777199)

[Figure S11 22](#_Toc124777200)

[Figure S12 23](#_Toc124777201)

[Figure S13 24](#_Toc124777202)

[Figure S14 25](#_Toc124777203)

[Figure S15 26](#_Toc124777204)

[Figure S16 27](#_Toc124777205)

[Figure S17 28](#_Toc124777206)

[Figure S18 29](#_Toc124777207)

[Figure S19 30](C:\\Users\\abe.cullom\\Desktop\\WR Edits\\SI_Cullom_CMPRPipeDis_230110.docx" \l "_Toc124777208)

[References 31](#_Toc124777209)

## Section SI-1. Influent water preparation

The influent water was prepared with Blacksburg, VA tap water that was breakpoint chlorinated to de-chloraminate and destroy residual ammonia, dosed with sodium thiosulfate to remove residual chlorine, adjusted to pH 7.5 using 1M hydrochloric acid, and bubbled to raise dissolved oxygen (DO) to ~8 mg/L. At the beginning of Phase 1, after the pipes had been sufficiently colonized and aged, the influent water was pasteurized to remove residual municipal disinfectant residual and avoid use of sodium thiosulfate at the starting point for disinfectant dosing. Pasteurization also served to minimize influent microbes as would occur in a typical storage water heater operated at 60° C. [1]. After dosing disinfectants into the influent waters, pH levels were adjusted back to 7.5 before use.

## Section SI-2. Disinfectant preparation

A 5,000 mg/L free chlorine stock solution was prepared from DI water and 7.5% bleach (Clorox, Alpharetta, GA), and a 1,000 mg/L stock solution of monochloramine was generated with the free chlorine stock solution and a 1400 mg/L ammonia stock solution from 14.8 N ammonia hydroxide (Fisher Scientific, Waltham, MA). Chlorine dioxide was prepared for each water change by dosing 2 L of prepared stock water with a Potable Aqua chlorine dioxide water purification tablet (Wisconsin Pharmacal Company, Jackson, WI). A 100:10 mg/L Cu:Ag CSI dosing solution was created by combining copper sulfate pentahydrate (Fisher Scientific, Waltham, MA) and silver nitrate (Alfa Aesar, Tewksbury, MA) in DI water. Chlorine, monochloramine, and chlorine dioxide were measured using a Hach DR5000 spectrophotometer (Loveland, CO) with Methods 8021, 10171, and 10126, respectively. Copper and silver concentration levels were measured using inductively coupled plasma mass spectroscopy (ICP-MS) (iCAP RQ ICP-MS; Thermo Fisher Scientific, Waltham, MA). All ICP-MS samples were acidified with 2% v/v nitric acid before measurement.

## Section SI-3. qPCR methods and quality control

Total bacteria, *P. aeruginosa*, and *A. baumannii* were molecularly quantified using qPCR assays targeting bacterial 16S rRNA gene [2], the *oprL* gene [3] the 16S-23S rRNA intergenic spacer (ABITS) [4], respectively. Evagreen assays were performed with SsoFast EvaGreen Supermix (BioRad, Hercules, CA) and probe assays with SsoAdvanced Universal Probes Supermix (BioRad, Hercules, CA). All assays were carried out at 1:10 sample dilutions to minimize metal inhibition using a with analytical triplicates and nuclease-free water (G-Biosciences, St. Louis, MO) as negative controls. See Table S1 for additional details. A standard curve was generated using dilutions of IDT gBlocks (Coralville, IA) of the target sequence in nuclease-free water at 10^1^-10^7^ gene copies/mL for *oprL* and ABITS and 10^2^-10^8^ gene copies/mL for the 16S rRNA gene. The quantification limit was designated by the lowest amplified standard, and only standard curves with R^2^>0.97 were accepted. For data analysis, below quantification limit data were set to one half of the lowest standard in the qPCR standard curve and below detection limit data were set to 0.

## Section SI-4. Software packages used in data analysis

**Data formatting:**

tidyverse [5] and readxl [6]

**Statistical testing and visualization:**

dunn.test [7], rcompanion [8], and gridExtra [9]

## Section SI-5. Disinfectant decay and half-life calculation

During the highest dose period (Week 28) the free chlorine, monochloramine, and chlorine dioxide concentrations were monitored at 0, 2, 19, and 43 hours stagnation time using the methods described above. A second trial with higher time-resolution was performed in conditions where disinfectants decayed rapidly the following water change. After confirming first-order decay kinetics were appropriate using generalized linear regression, disinfectant half-lives were calculated from the resulting models.

## Section SI-6. Discussion of water chemistry findings

Each pipe material was found to present inherent advantages and disadvantages in terms of water quality and conduciveness to *P. aeruginosa* and *A. baumannii* populations. In particular, pH is a key driver of microbial ecology and also dictates the efficacy of disinfectants, either as by governing weak acid chemistry (e.g., chlorine-based disinfectants) [10] or solubility of metals (e.g., copper, silver) [11]. Bulk water pH measurements were consistent with metallic pipe corrosion resulting in elevated pH and correspondingly decreased potency of disinfectants [12–14]. Addition of disinfectant can also act to elevate pH by reducing bacterial respiration and corresponding production of acidic CO_2_ [15]. Elevated pH can also enhance disinfectant-induced corrosion [16].

While both OPs investigated in this study are aerobic, they have been reported to be tolerant of relatively low-DO conditions or even periodic anaerobic conditions [17]. In particular, iron was found to deplete DO to the greatest extent relative to other pipe materials, with a typical effluent concentration of ~2 mg/L (Fig S4). TOC is also known to be a key factor limiting regrowth in drinking water systems [1]. Notably, chlorine-based disinfectants induced the undesirable effect of increasing TOC in the CMPRs, especially in the initial stage of chlorine application, which is consistent with prior research demonstrating chlorine-induced TOC release from plastics [18].

## Section SI-7. OPs correlation with water quality parameters

In Phase 2, DO was positively correlated with *P. aeruginosa* gene copies (Spearman’s rho=0.74, p=0.003) and *P. aeruginosa* relative abundance (rho=0.67, p=0.01) in disinfectant-free PVC CMPRs and all PVC CMPRs when considering all disinfectant conditions in PVC via CCA (Fig S19). DO further correlated with *P. aeruginosa* relative abundance in disinfectant-free iron-PVC CMPRs (rho=0.60, p=0.04) and PVC CMPRs across all disinfectant conditions (rho=0.43, p=0.004).

Total bacteria were positively correlated with *A. baumannii* gene copies in copper-PVC (Spearman’s rho=0.84, p<0.0001) and iron-PVC CMPRs (rho=0.86, p<0.0001) across all disinfectant conditions in Phase 2, but not *P. aeruginosa*. TOC was negatively correlated with *P. aeruginosa* relative abundance across all PVC CMPRs in Phases 0 and 1 (rho=-0.46, p=0.02), but positively so in Phase 2 (rho=0.42, p=0.004). Additionally, TOC was appeared negatively correlated with *P. aeruginosa* gene copies in PVC and iron-PVC via CCA. Total copper was negatively correlated with culturable *P. aeruginosa* in copper-PVC CMPRs in Phases 0 and 1 (rho=-0.49, p=0.01) and overall via CCA, agreeing with the observed high effectiveness of CSI for culturable *P. aeruginosa* control. Total iron was positively correlated with culturable *P. aeruginosa* in iron-PVC CMPRs receiving chlorine dioxide (rho=0.82, p=0.04) and overall via CCA, as well as with *A. baumannii* gene copies across all disinfectant conditions (rho=0.40, p=0.02).

Disinfectant doses were only correlated negatively with culturable *P. aeruginosa* levels in PVC and copper-PVC CMPRs that received CSI (Spearman’s rho=-0.55, -0.48 and p=0.009, 0.03, respectively), and were not negatively correlated with *P. aeruginosa* gene copies in any context. Chlorine (rho=-0.77, p<0.0001), chlorine dioxide (rho=-0.63, p=0.002), and CSI (rho=-0.70, p=0.0004) were negatively correlated with culturable *A. baumannii*

## Section SI-8. Comparison of pipe corrosion results to previous studies

All three oxidizing disinfectants (monochloramine, chlorine and chlorine dioxide) increased corrosion of iron pipes when dosed at high levels, but the extreme observed in chlorine dioxide were likely influenced by the chloride and sulfate salts present in the tablets used to generate chlorine dioxide (Table S4). In contrast to our study, Eisnor *et al.* [19] found that monochloramine released the most iron from cast iron pipes, and chlorine dioxide released the least. Notably, the Eisnor *et al.* study generated chlorine dioxide via a method (sodium hypochlorite and sulfuric acid) [20] that should theoretically add far less excess chloride and sulfate than was the case for the present study and also used a cast-iron pipe that was over 100 years old. Zhang *et al.* [21] observed higher iron corrosion rates with chlorine dioxide than free chlorine, but differences were more modest (Table S4). Despite the dose of chlorine dioxide in the present study being a fifth of the free chlorine concentration, chlorine dioxide dosing was associated with much greater iron corrosion relative to the Zhang et al. study, where roughly equivalent doses of chlorine and chlorine dioxide were applied.

## Section SI-9. Comparison of culture and qPCR methods

Overall, correlations between levels as measured by culture and molecular methods were significant but modest for both OPs (Spearman’s rho=0.28, p<0.0001 for *P. aeruginosa*, rho=0.50, p<0.0001 for *A. baumannii*). Notwithstanding, how indicative culturable levels were of gene copies were highly context dependent (Fig S18) When examining disinfectant-free CMPRs on a material-by-material basis, correlations were weakest for both OPs in iron CMPRs, and not significant for *P. aeruginosa* (p=0.053). When examining data from Phase 2 on a disinfectant-by-disinfectant basis, *P. aeruginosa* culturable levels were only correlated with gene copies in monochloramine CMPRs (Spearman’s rho=0.54, p=0.003), but in all disinfectant conditions for *A. baumannii*. In general, correlations were poorer where there were lower levels of culturable OPs, possibly due to a lower detection limit via culture methods, and correlations were stronger for *A. baumannii*.

While it was encouraging that both methods generally indicated the same overall trends in terms of the response of the target OPs to the disinfectants, culture counts tended to be reduced by much lower doses of disinfectant than gene copies and correlations between the two measurements were weak in instances where culture counts were low. For example, the dose of CSI required to significantly reduce *P. aeruginosa* cell counts and gene copies in copper-PVC CMPRs varied by an order of magnitude (0.025:0.0025 mg/L Cu:Ag for culturable levels, 0.25:0.025 mg/L for gene copy levels). While qPCR does not directly measure viability and so inevitably measures some inactivated cells, the CMPRs experienced each disinfectant dose for a minimum of 6 weeks before sampling, allowing cells susceptible to the higher dose sufficient time to be inactivated and degraded or removed from the bulk water during the thrice weekly 100% water changes. Additionally, qPCR-culture discrepancies were not consistent and generally widened with greater disinfectant doses, with that for *P. aeruginosa* in PVC CMPRs increasing from 0.35 logs higher by qPCR during week 6 to 2.30 logs by week 30, implying that culture counts genuinely overestimated the extent of total inactivation. These discrepancies were also particularly high in contexts where CSI was applied. *P. aeruginosa* is known to enter a viable-but-not-culturable state in response to residual disinfectants, particularly free chlorine [22] and copper [22,23], while this has not been reported for *A. baumannii*. This, along with the trend of qPCR-culture correlations being weaker for *P. aeruginosa* than *A. baumannii*, also seems to indicate that culture counts overestimated inactivation in some cases by missing viable-but-not-culturable cells.

## Section SI-10. Disinfectant efficacy compared to previous studies

Previous bench-scale studies have generally indicated more effective inactivation of the two OPs examined in this study with the same disinfectants, highlighting the importance of comprehensive studies that are more representative of the complexities encountered in premise plumbing. Grobe *et al.* [24] observed 0.39 to >5 log reductions in *P. aeruginosa* cell counts numbers after only 5 minutes of exposure to an initial dose of 0.45 mg/L free chlorine. This was approximately half the dose in the week 19-24 period of this study, after which we observed no statistically significant reduction in *P. aeruginosa* gene copies or cell counts. Bédard *et al.* [25] summarized chlorine, monochloramine, and chlorine dioxide results from bench-scale studies, which similarly yielded more optimistic estimates of the disinfectant efficacy than the present study. Wang *et al.* [26] observed a >5 log reduction in *A. baumannii* cell counts after 60 minutes of exposure to a 1 mg/L free chlorine dose, whereas, here, limited or no reductions in *A. baumannii* gene copies or cell counts were observed at a similar dose. It should be noted that some of these discrepancies may partially be explained by differences between reductions measured by culture counts and qPCR (Section SI-9).

in PVC. Interestingly, these disinfectants as well as monochloramine in PVC and free chlorine in both copper-PVC and iron-PVC were also associated with reductions in *A. baumannii* gene copies.

## Section SI-11. Analysis of temperature data

Target temperatures were achieved across all CMPRs, averaging 37.6 ± 1.1°C (mean ± standard deviation). Target temperatures were maintained over the duration of the experiment (Fig S2), and were 0.2°C lower within metallic CMPRs (One-way ANOVA and Tukey multiple comparisons test, p<0.05) possibly as a result of heat loss from metallic pipe sections (average temperature in PVC: 37.7°C, copper-PVC and iron-PVC: 37.5°C). When CMPRs were grouped by disinfectant and pipe type in Phase 2, a difference was observed in 19 out of 30 possible comparisons (One-way ANOVA and Tukey multiple comparisons test, p<0.05) and generally indicated that PVC and iron-PVC CMPRs were slightly warmer when CSI was dosed, while copper-PVC CMPRs were warmer when CSI, monochloramine and chlorine dioxide were dosed. This is possibly because locations in the center of the water bath were slightly warmer (Fig S3). However, the average temperatures of all CMPRs were within the ideal growth range of the two target OPs and ± 1.5°C across all pipes.

## SI Tables

Table S1. Quantitative chain polymerase chain reaction (qPCR) run parameters and primer/probe sequences

| **Run parameters** |  |  |  |  |  |  |  |  |
| --- | --- | --- | --- | --- | --- | --- | --- | --- |
| **Target** | **Initial Denaturation** | | **Annealing/Extension** | | | **Melt Curve** | | |
|  | **Temp** | **Time (min)** | **Temp (°C)** | **Time (min** | **Cycles** | **Temp (°C)** | | |
| **16S rNA Gene** | 98 | 2 | 55 | 5 | 40 | 65 | - | 95 |
| ***oprL*** | 95 | 2 | 58.3 | 15 | 40 | NA | | |
| **ABITS** | 98 | 2 | 55 | 5 | 40 | 65 | - | 95 |

| **Primers/Probe Sequences** | | |  |
| --- | --- | --- | --- |
| **Target** | **Forward** | **Reverse** | **Probe** |
| **16S rNA gene** | CGGTGAATACGTTCYCGG | GGWTACCTTGTTACGACTT | NA |
| ***oprL*** | AACAGCGGTGCCGTTGAC | GTCGGAGCTGTCGTACTCGAA | /56-FAM/TGAGCGACGAAGCC/3BHQ_1/ |
| **ABITS** | CATTATCACGGTAATTAGTG | AGAGCACTGTGCACTTAAG | NA |

| **Target** | **gBlock Sequences** |
| --- | --- |
| **16S rNA gene** | TGCCACGGTGAATACGTTCCCGGGCCTTGTACACACCGCCCGTCACACCATGGGAGTGGGTTGCAAAAGAAGTAGGTAGCTTAACCTTCGGGAGGGCGCTTACCACTTTGTGATTCATGACTGGGGTGAAGTCGTAACAAGGTAACCGTAGG |
| ***oprL*** | GCATGGCTTCCGGCTTCAGGTCGGAGCTGTCGTACTCGAAGTAGAAGGTGGTGATCGCACGCAGAGCGGCTTCGTCGCTCAGGCTGCCGTCAACGGCACCGCTGTTGGCGCCATAGCCTGCGTTC |
| **ABITS** | GATTTCATTATCACGGTAATTAGTGTGATCTGACGAAGACACATTAACTCATTAACAGATTGGCAAAATTGAGTCTGAAATAAATTGTTCACTCAAGAGTTTAGGTTAAGCAATTAATCTAGATGAATTGAGAACTAGCAAATTAACTGAATCAAGCGTTTTGGTATGTGAATTTAGATTGAAGCTGTACAGTGCTTAAGTGCACAGTGCTCTAAACT |

Table S2. Decay of Oxidizing Disinfectants in CMPRs at the Highest Dose

|  | **Calculated Half-Life Based on First-Order Kinetics (Hours)** | | | **Detectable After 43 Hours?** | | |
| --- | --- | --- | --- | --- | --- | --- |
| **Disinfectant** | PVC | Copper-PVC | Iron-PVC | PVC | Copper-PVC | Iron-PVC |
| **Cl_2_** | 57 | 16 | 4.9 | Yes | Yes | No |
| **NH_2_Cl** | 95 | 124 | 5.9 | Yes | Yes | No |
| **ClO_2_** | 24 | 8.5 | 11 | Yes | No | No |

Table S3. *A. baumannii* and *P. aeruginosa* present in effluent at the end of Phases 1 and 2 as a percentage of the number of bacteria inoculated.

| **­_­­_** | ***A. baumannii*** | | | | | |
| --- | --- | --- | --- | --- | --- | --- |
|  | **PVC** | | **Copper** | | **Iron** | |
|  | Phase 1 | Phase 2 | Phase 1 | Phase 2 | Phase 1 | Phase 2 |
| **Control** | 7.91% | 10.53% | 0.00% | 18.73% | 5.74% | 1.06% |
| **Free Chlorine** | 22.99% | 7.84% | 0.00% | 0.64% | 9.60% | 0.99% |
| **Monochloramine** | 28.81% | 3.80% | 19.67% | 15.86% | 5.34% | 44.30% |
| **Chlorine Dioxide** | 43.02% | 0.00% | 20.66% | 2.17% | 3.14% | 7.43% |
| **Copper-Silver** | 0.86% | 0.00% | 4.99% | 0.00% | 0.23% | 0.00% |
|  | ***P. aeruginosa*** | | | | | |
|  | **PVC** | | **Copper** | | **Iron** | |
|  | Phase 1 | Phase 2 | Phase 1 | Phase 2 | Phase 1 | Phase 2 |
| **Control** | 8.93% | 29.64% | 7.61% | 52.70% | 2.05% | 2.97% |
| **Free Chlorine** | 27.30% | 22.07% | 2.40% | 1.81% | 0.19% | 2.80% |
| **Monochloramine** | 14.75% | 10.70% | 8.50% | 44.63% | 1.50% | 124.65% |
| **Chlorine Dioxide** | 4.28% | 0.00% | 5.12% | 6.09% | 0.47% | 20.91% |
| **Copper-Silver** | 1.46% | 0.00% | 0.00% | 0.00% | 0.05% | 0.00% |

Table S4**.** Comparison of chloride and sulfate addition resulting from chlorine dioxide and free chlorine dosing, scaled by ppm of either ClO_2_ or free Cl_2_. Values from *Eisnor et al.* are calculated based on assumed, typical reaction efficiency, as these authors used analytical grade reagents for disinfectant generation that should not contain excess chloride or sulfate.

| **Additional (ppm/ppm disinfectant)** | | **ClO_2_** | **Free Chlorine** | **Fold Difference** |  |  |  |
| --- | --- | --- | --- | --- | --- | --- | --- |
| **Cl^-^** | This study | 34.6 | 4.83 | 7.16 |  |  |  |
|  | Eisnor *et al.*^a^ | 1.62 | 0.51 | 3.17 |  |  |  |
|  | Zhang *et al.*^b^ | 3.58 | 6.68 | 0.54 |  |  |  |
| **SO4^2-^** | This study^c^ | 104 | 0.22 | 465 |  |  |  |
|  | Eisnor *et al.*^a^ | 2.19 | 0 | - |  |  |  |
|  | Zhang *et al.*^b^ | -2.15 | -1.62 | - |  |  |  |
| a. Assuming reaction efficiencies of 98% for chlorine and 65% for chlorine dioxide generation | | | | | | | |
| b. Average across 1, 2, and 4 mg/L conditions | | | |  |  |  |  |
| c. SO_4_ concentration calcuated from sulfur concentration | | | | |  |  |  |

## SI Figures

**
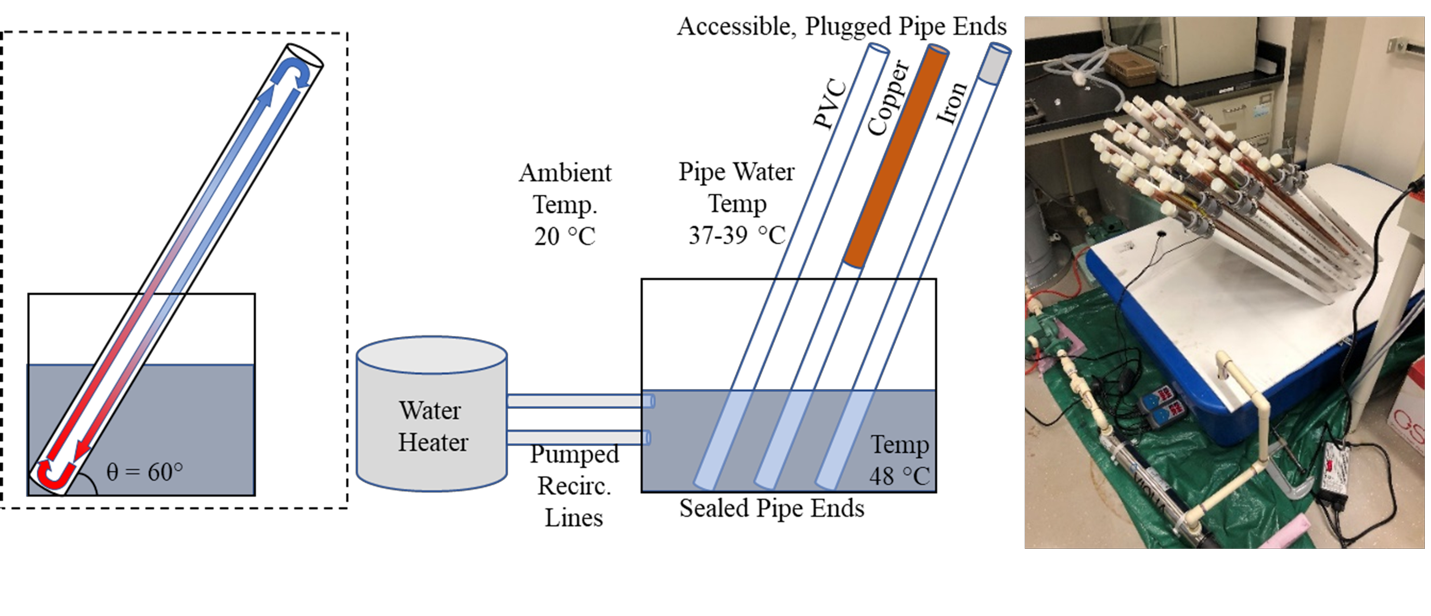
**

Figure S1**.** Convective mixing pipe reactor. Pipes were concentrated in the center to prevent temperature differences between conditions. The system occupies ~20 square feet of floor space and requires no connections to building plumbing. Figure from Spencer *et al.* [22].

Figure S2**.** Average differences in effluent temperature between disinfectant and disinfectant-free conditions by Phase. Average temperatures (°C) in the disinfectant-free (control) condition are displayed in gray. Error bars represent 95% non-parametric bootstrap confidence intervals (n=3, 18, 27, 3, for disinfectant conditions and n=6, 36, 54, 6 for disinfectant-free conditions in Phases 0, 1, 2, and 3, respectively).
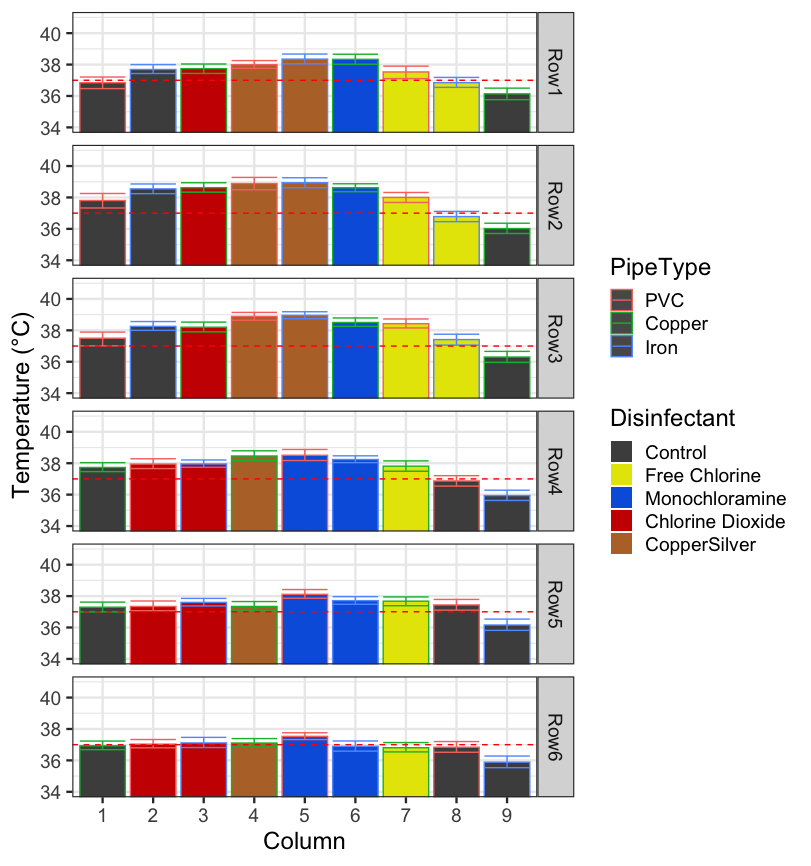


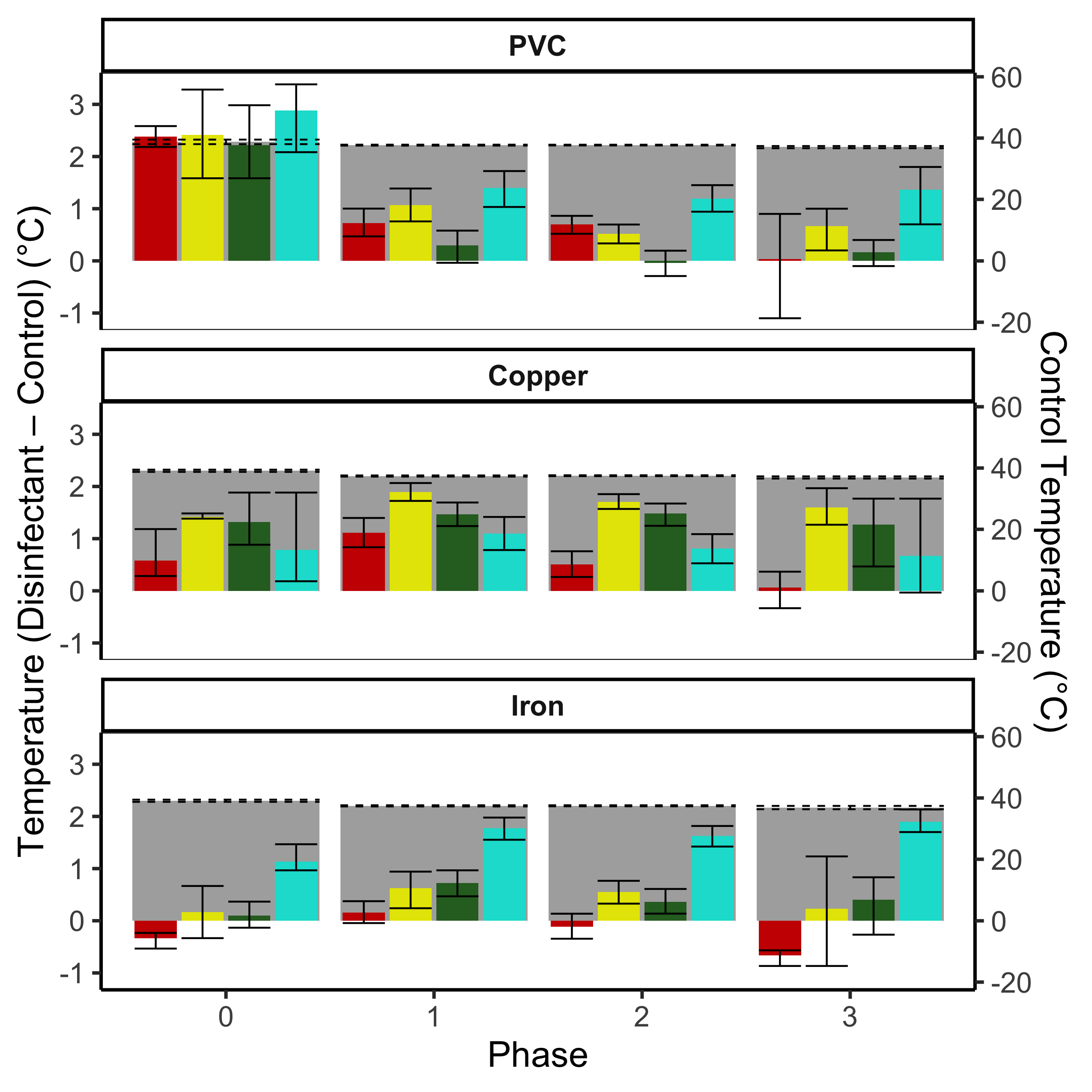

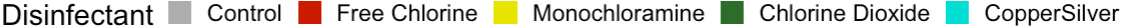


Figure S3. Average effluent temperatures based on pipe location. Error bars represent 95% non-parametric bootstrap confidence intervals, n=17. The dashed red line represents the target temperature of 37°C.


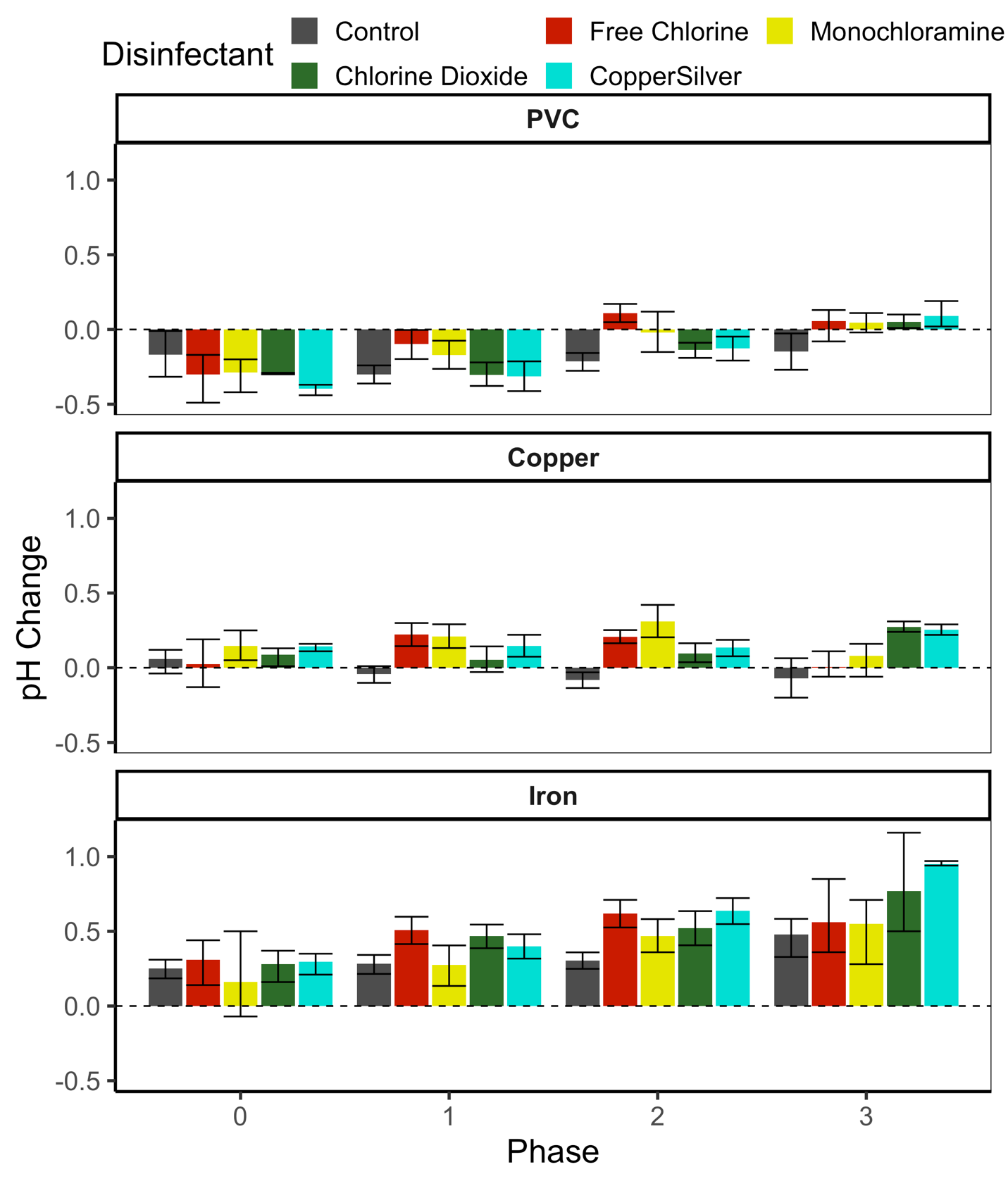


Figure S4**.** Effect of pipe material and disinfectant on effluent pH. Error bars represent 95% non-parametric bootstrap confidence intervals (n=3, 18, 27, 3, for disinfectant conditions and n=6, 36, 54, 6 for disinfectant-free conditions in Phases 0, 1, 2, and 3, respectively).


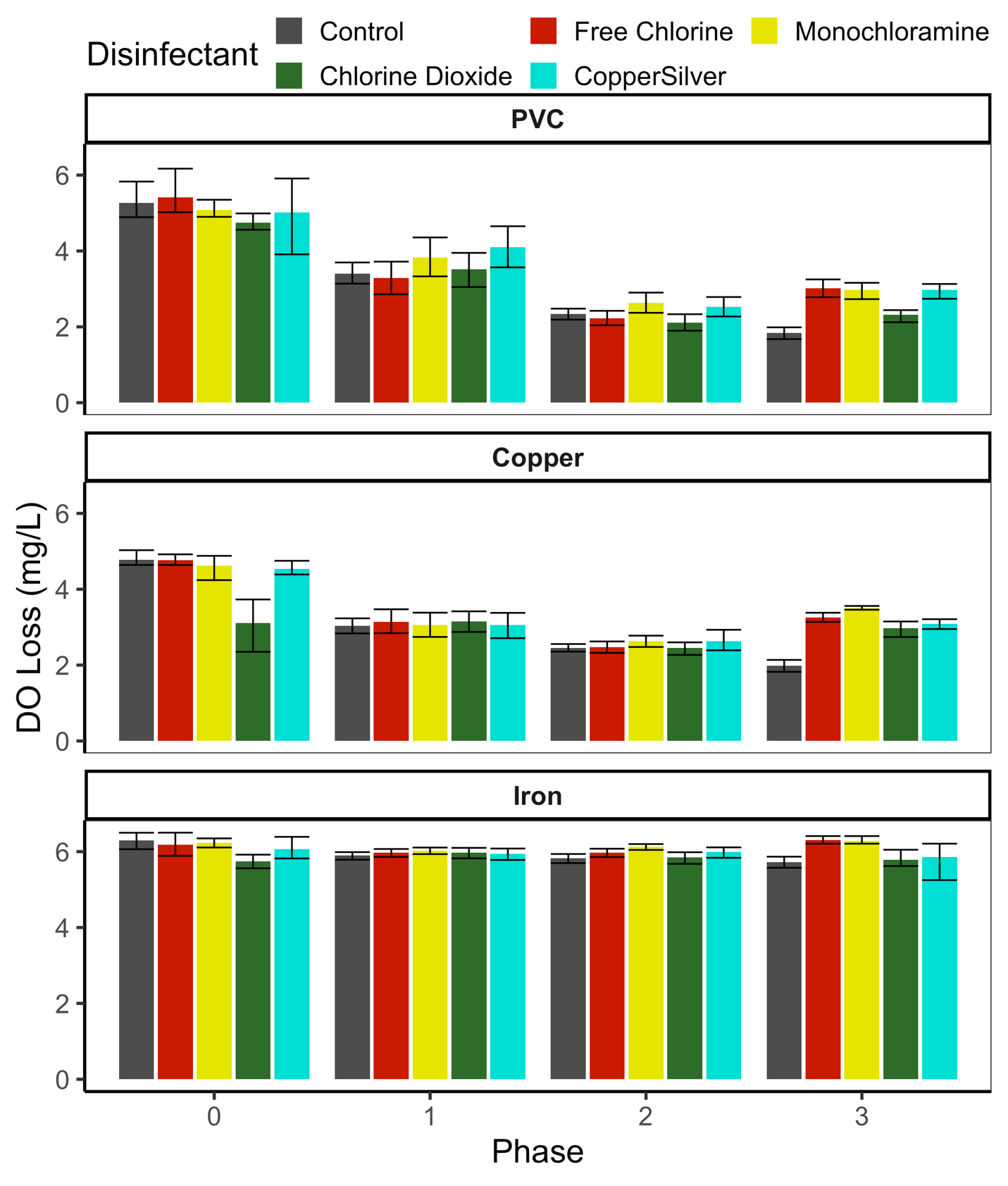


Figure S5**.** Effect of pipe material and disinfectant on DO loss in CMPR bulk water. Error bars represent 95% non-parametric bootstrap confidence intervals (n=3, 15, 27, 3, for disinfectant conditions and n=3, 30, 54, 6 for disinfectant-free conditions in Phases 0, 1, 2, and 3, respectively).


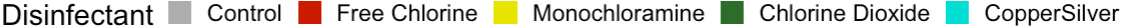

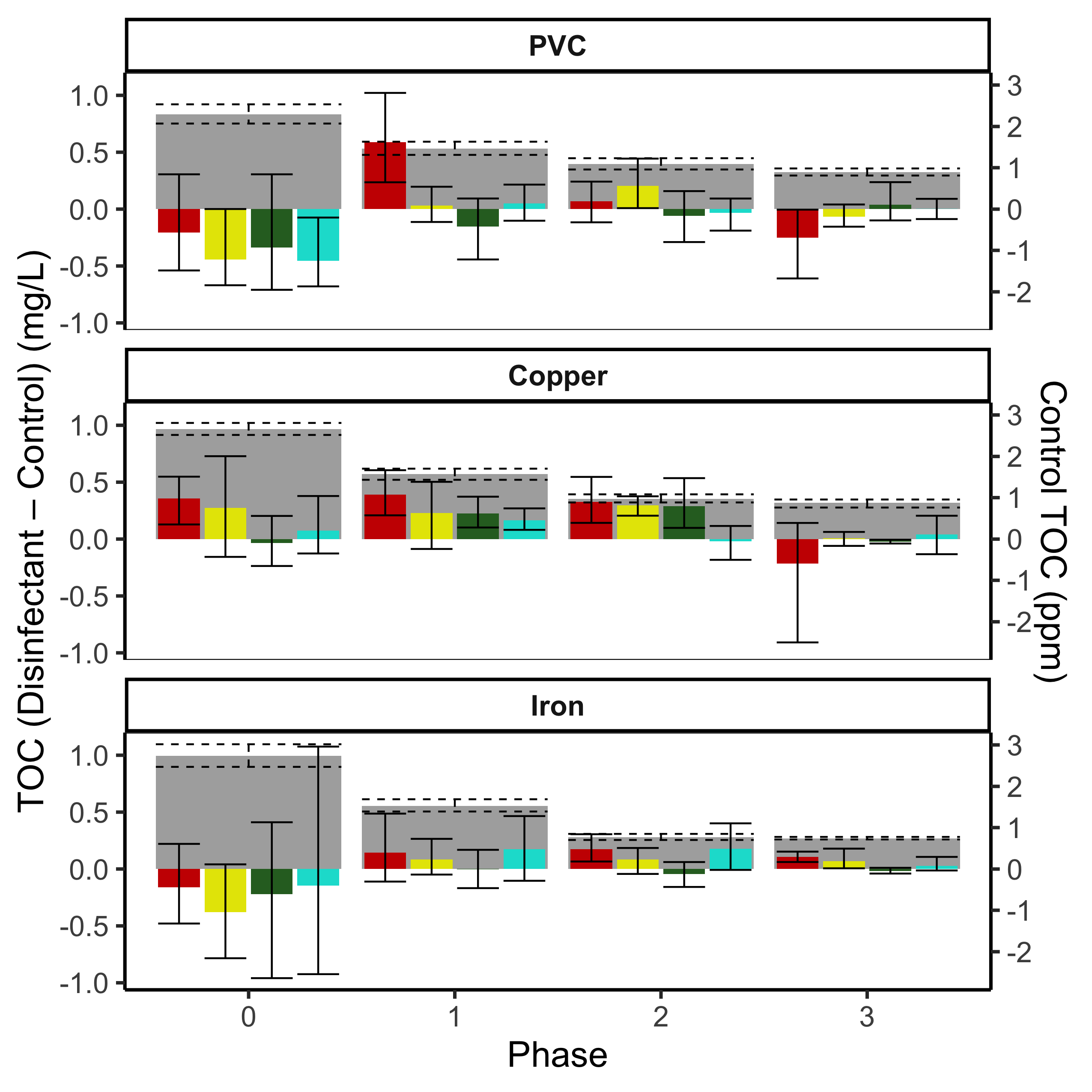


Figure S6**.** Average differences in total organic carbon (TOC) concentration between disinfectant and disinfectant-free conditions by Phase. Average TOC concentrations (mg/L) in the disinfectant-free (control) condition are displayed in gray. Error bars represent 95% non-parametric bootstrap confidence intervals (n=3, 18, 27, 3, for disinfectant conditions and n=6, 36, 54, 6 for disinfectant-free conditions in Phases 0, 1, 2, and 3, respectively).

Figure S7**.** Differences A) soluble and B) total phosphorus measurements via ICP-MS between disinfectant and disinfectant-free conditions at the end of Phase 0 (Week -1), Phase 1 (Week 11), and Phase 2 (Week 29). Average concentrations in parts per billion (ppb) in the disinfectant-free condition are displayed for each timepoint. Error bars represent range. Samples sizes are n=3 for CMPRs that received disinfectant, n=6 for controls.

**
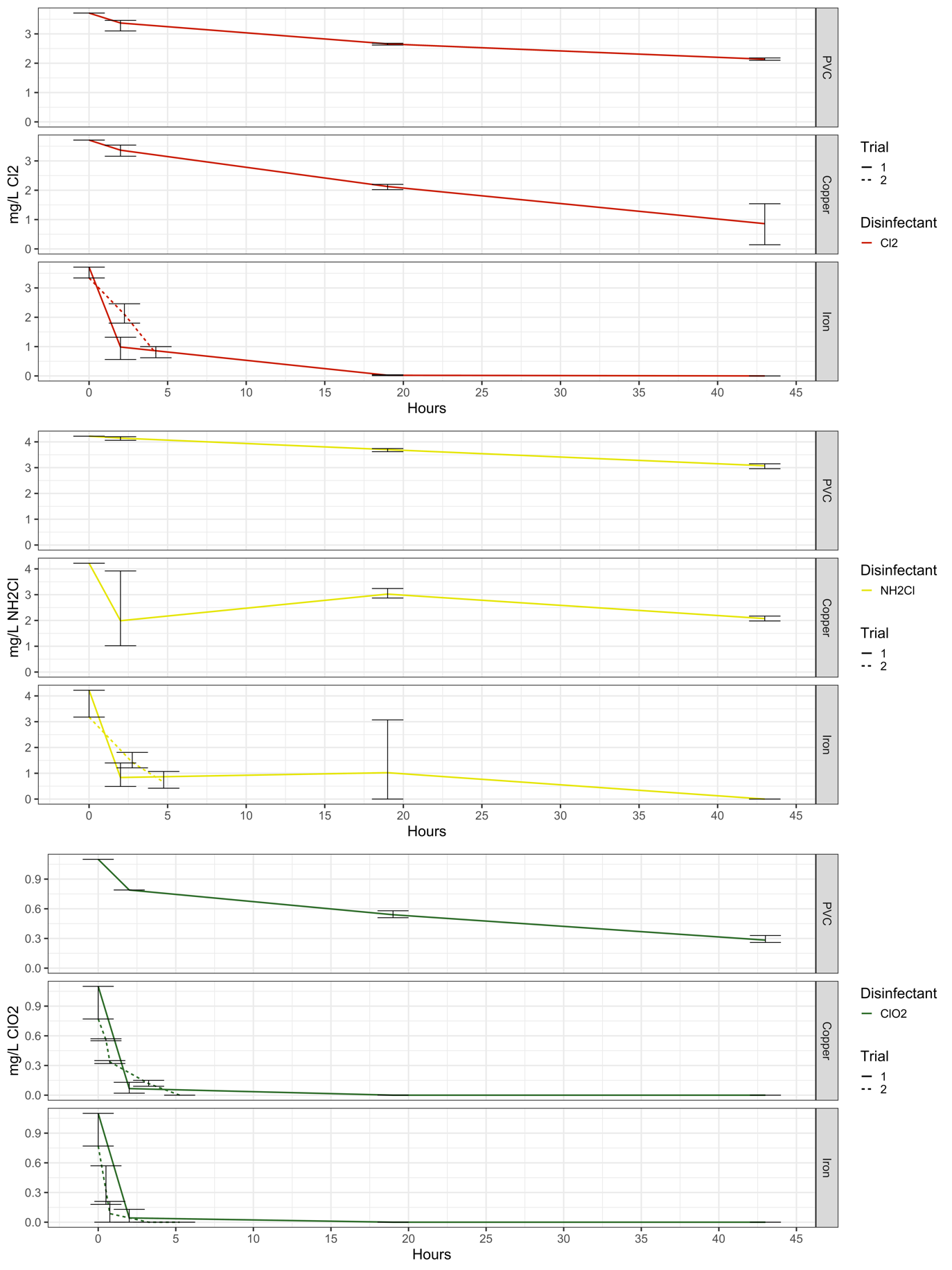
**

Figure S8**.** Residual decay measured at two timepoints during week 28 (Phase 2), with CMPRs receiving the highest disinfectant doses. Error bars represent range

Figure S9**.** Effects of pipe materials on bacterial targets in absence of disinfectant. Levels of A) total bacterial 16S rRNA gene copies, B) *P. aeruginosa* *oprL* gene copies and C) *A. baumannii* 16S-23S intergenic spacer copies. Letters denote groupings based on differences from post-Kruskal Wallis Dunn’s Test (p<0.05) Sample size is n=6.

**
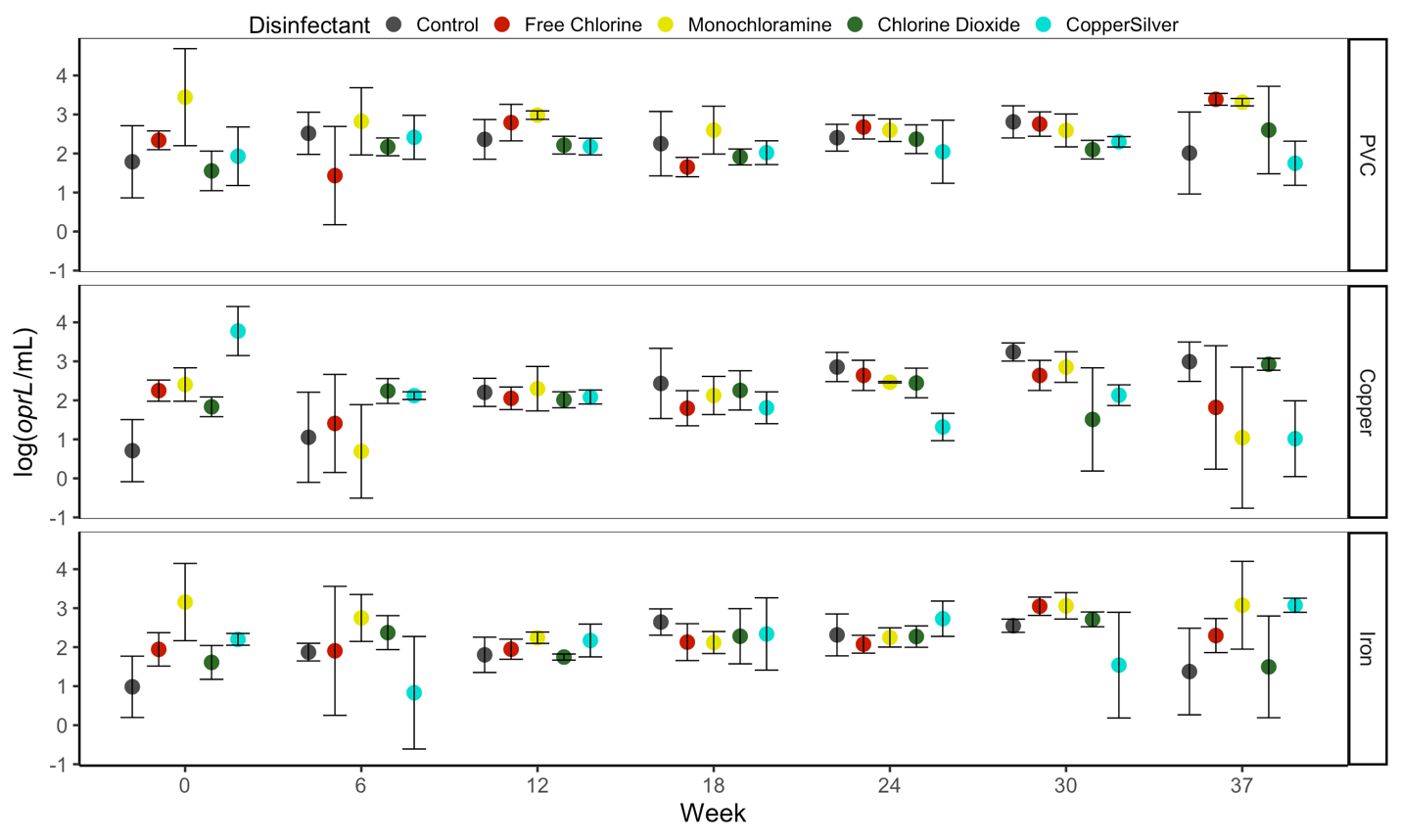

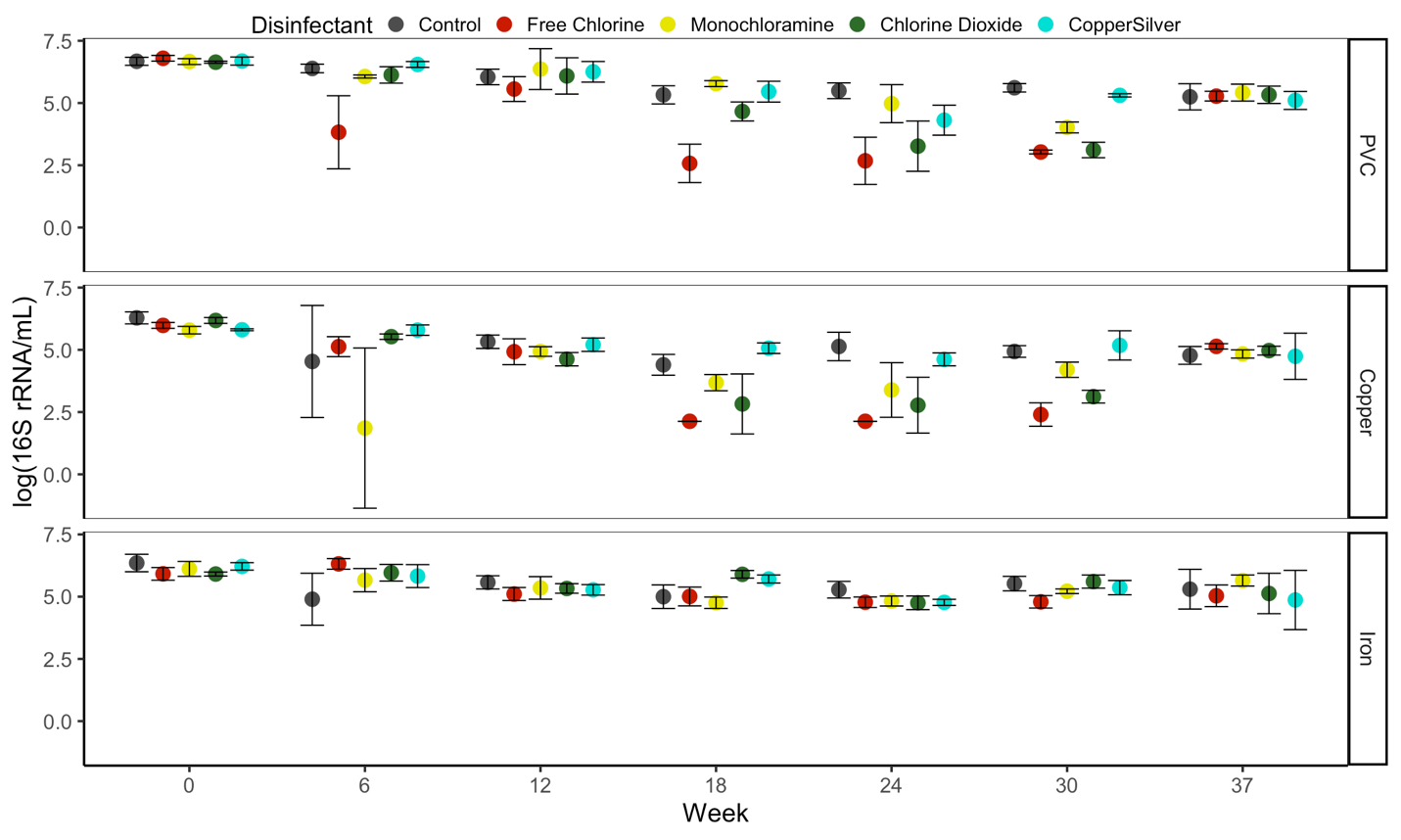
**

B)

A)

**
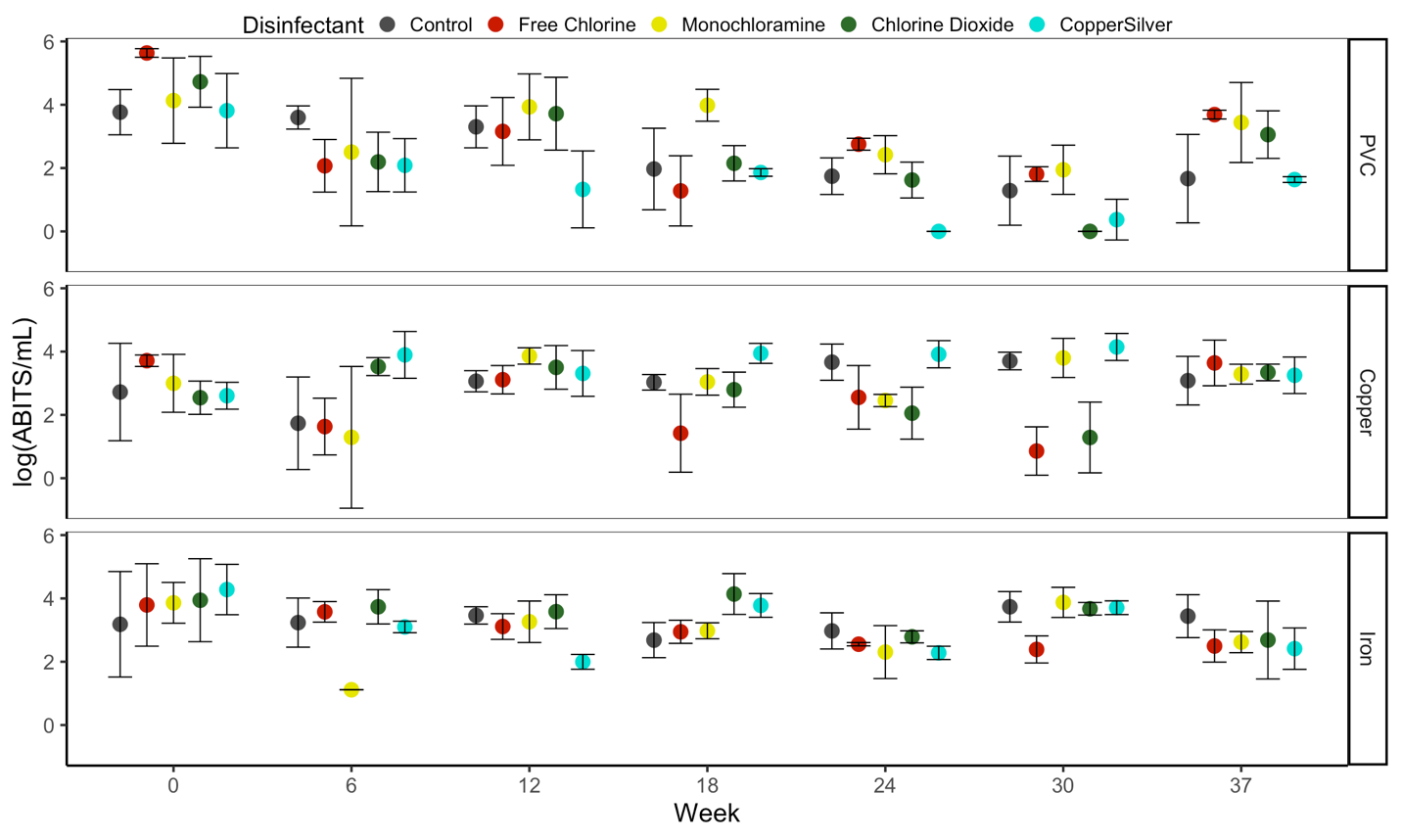
**

C)

Figure S10**.** Average levels of A) total bacteria (16S rRNA gene copy numbers), B) *P. aeruginosa* (*oprL* gene copy numbers), and C) *A. baumannii* (16S-23S rRNA gene intergenic spacer (ABITS) copy numbers) measured by qPCR in CMPR bulk water. Error bars indicate mean ± standard deviation. Samples sizes are n=3 for CMPRs that received disinfectant, n=6 for disinfectant-free controls.

Figure S11**.** Differences in culturable A) *P. aerguginosa* and B) *A. baumannii* levels between disinfectant and disinfectant-free conditions in CMPR effluent. Symbols indicate statistical significance of difference via Dunn’s Test (● = p>0.05, **▲** = p<0.05). Mean remaining disinfectant residual following stagnation as a percentage of the NPDWS or MCL are displayed for each condition. Samples sizes are n=3 for CMPRs that received disinfectant, n=6 for controls.


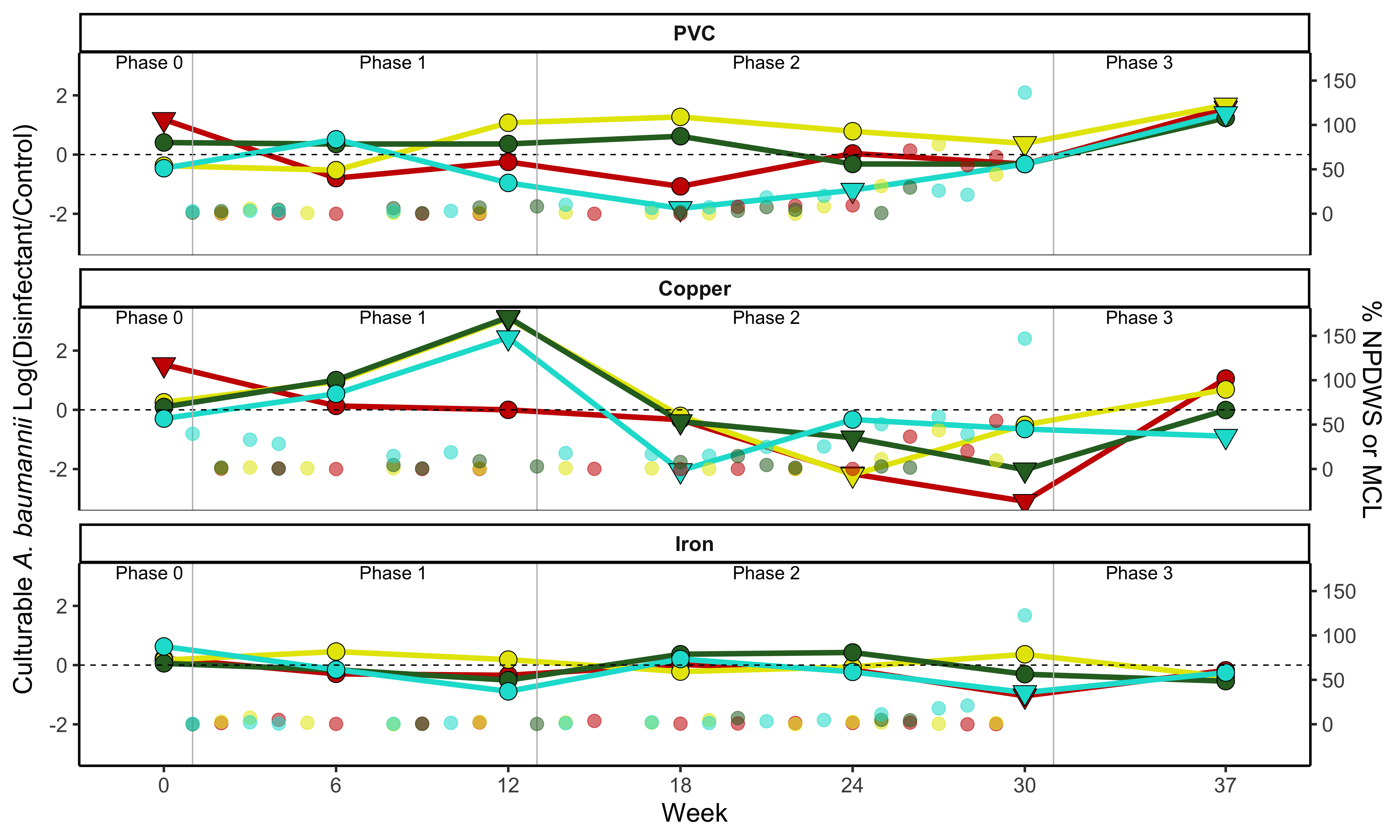

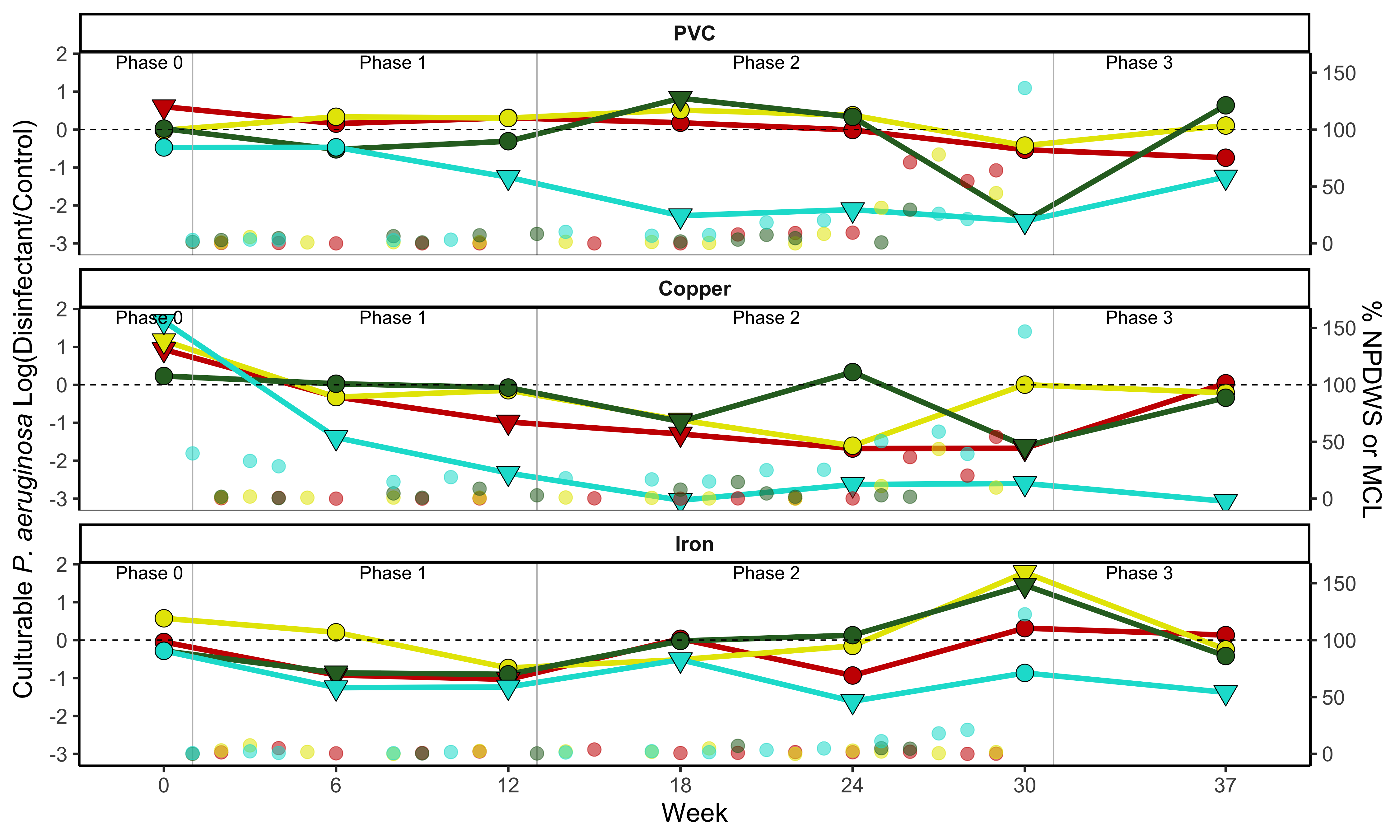


A)

B)


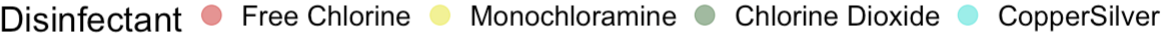


Measured disinfectant in effluent


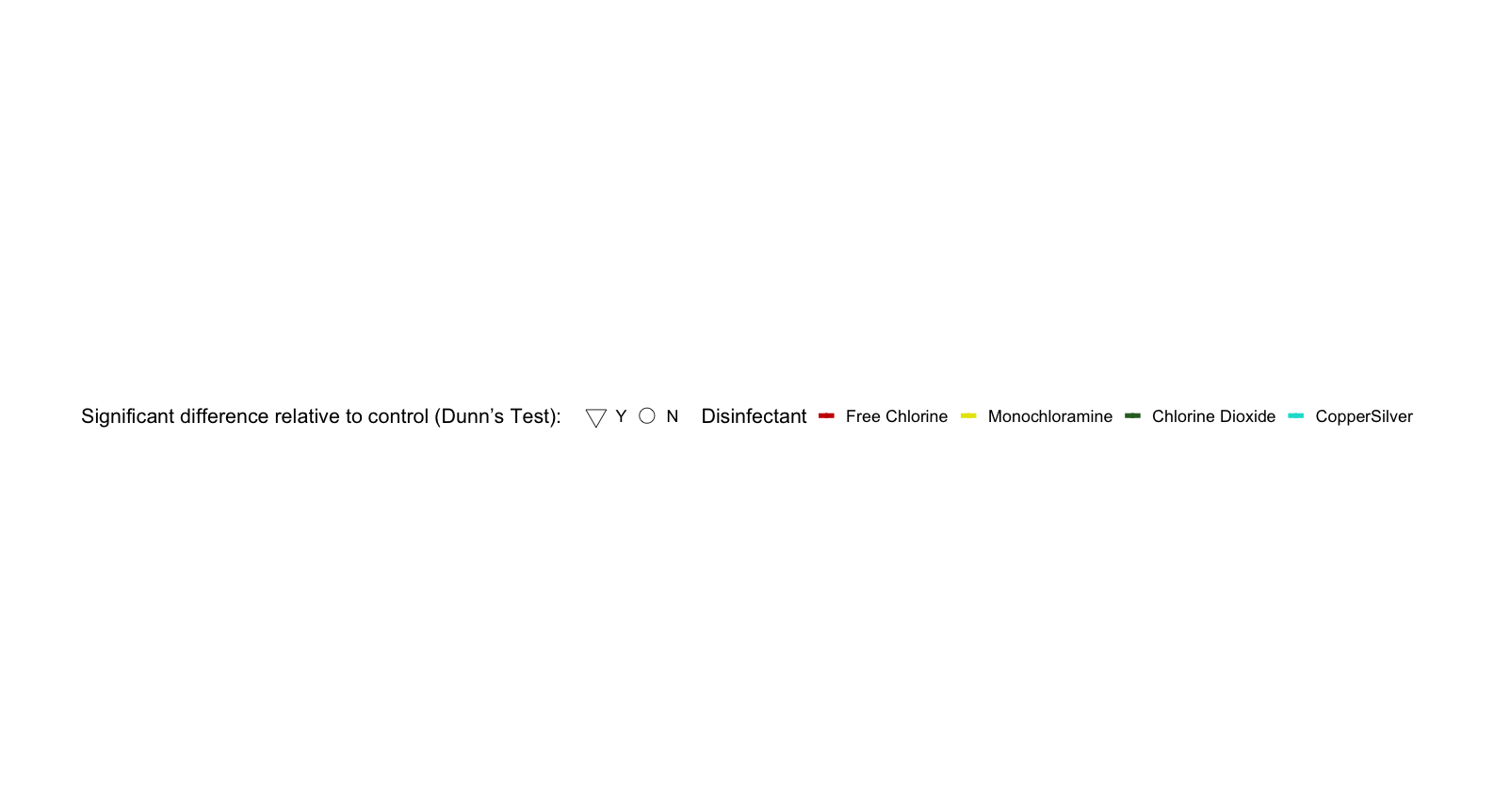

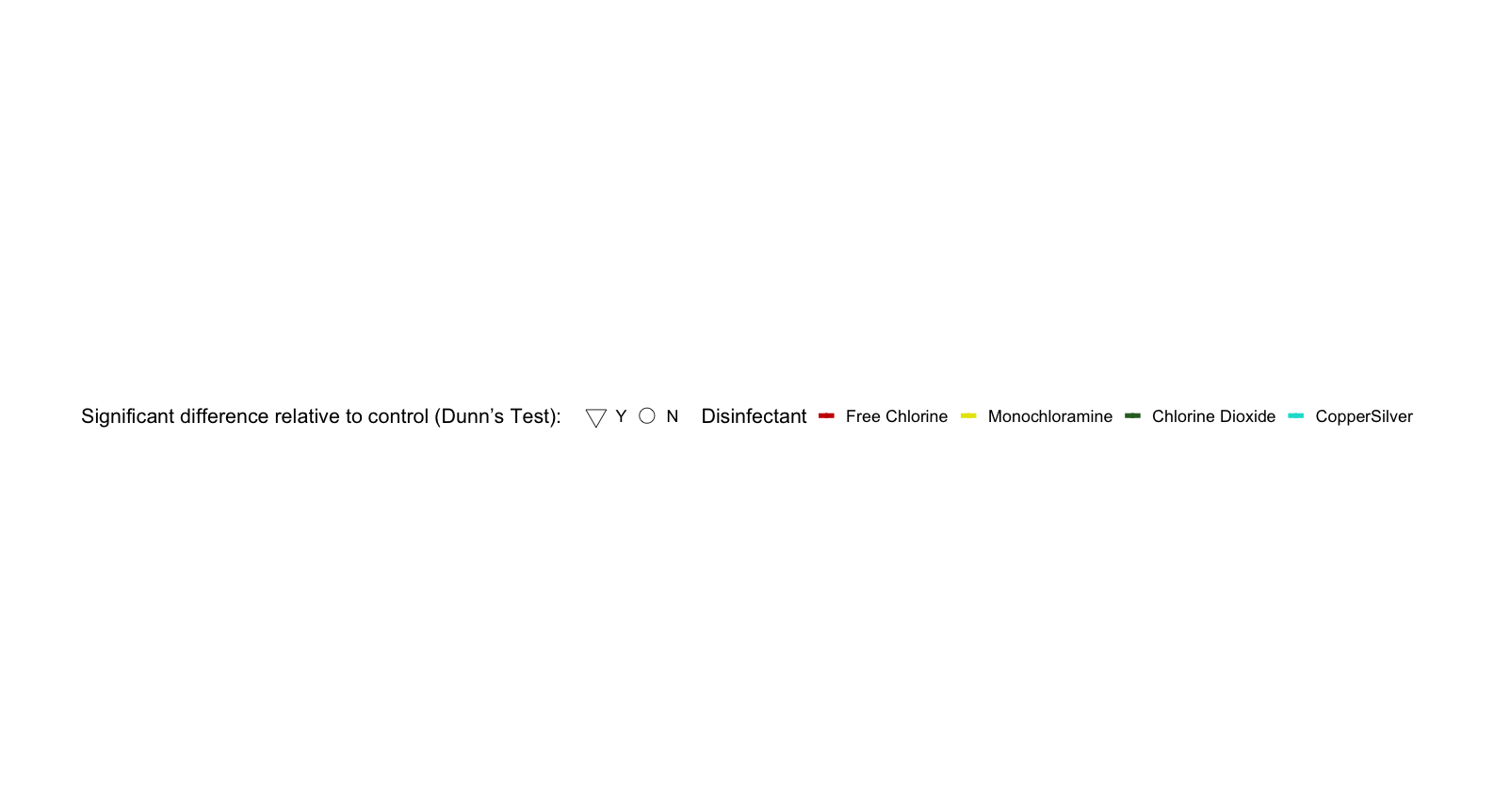


Disinfectant


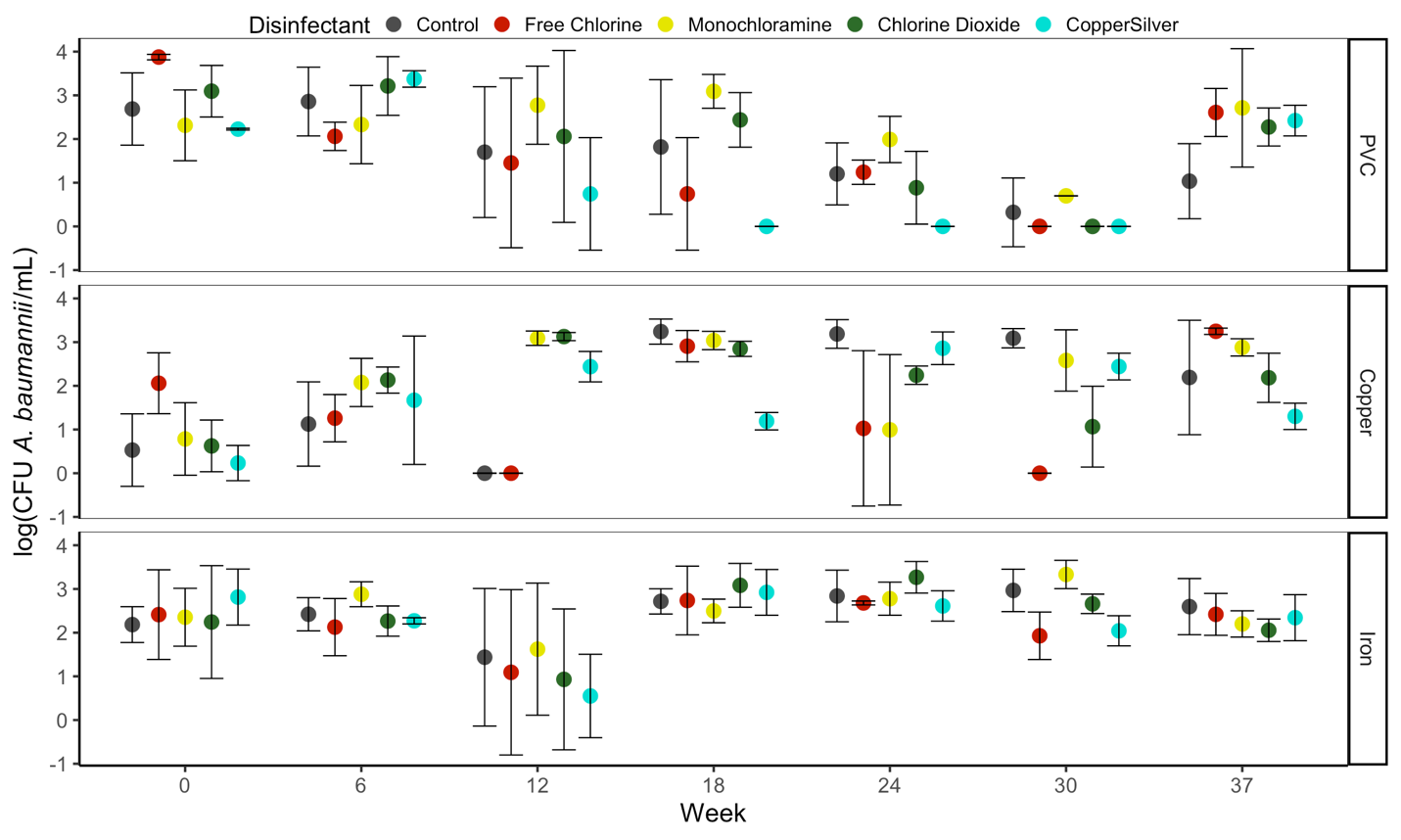

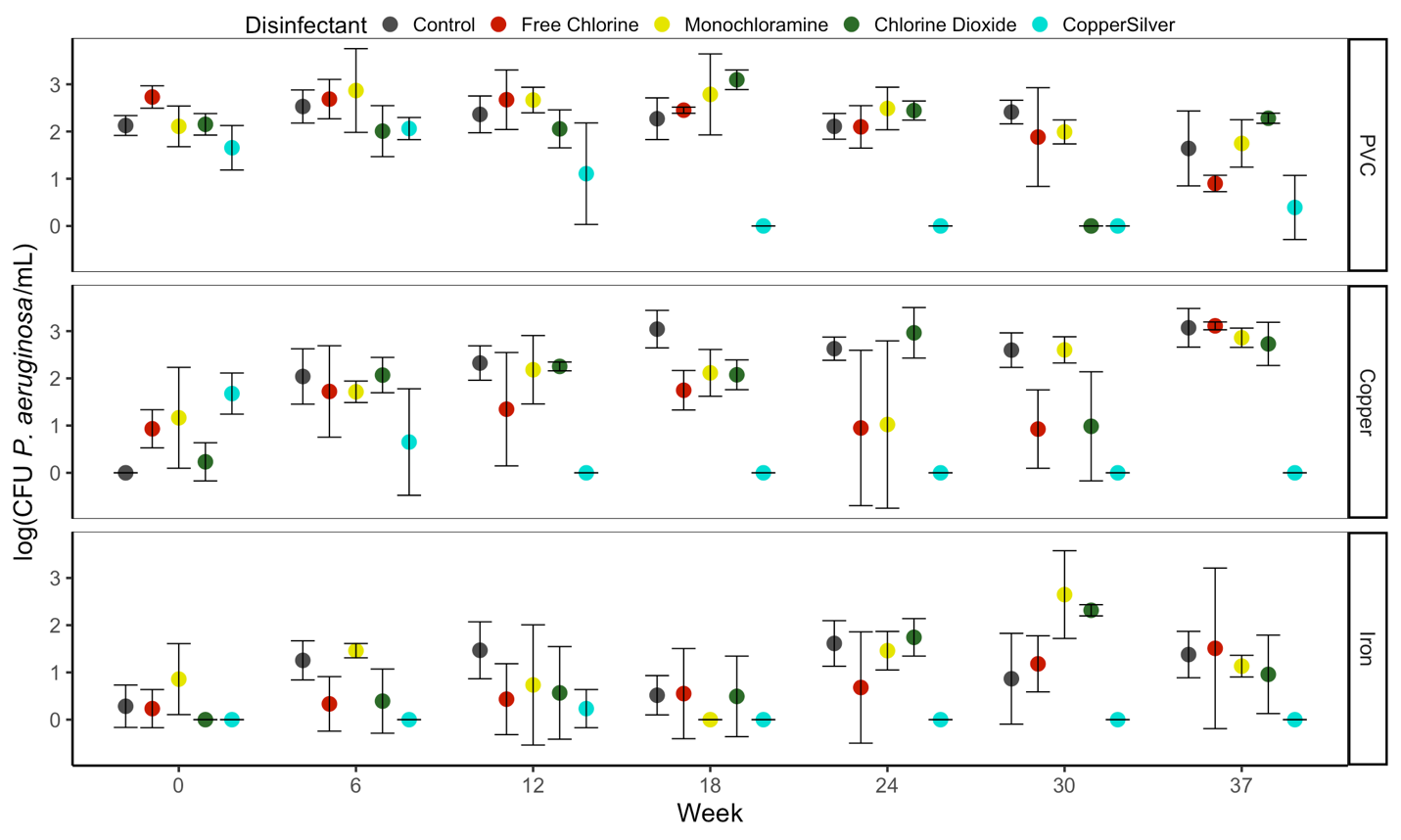
Figure S12**.** Average levels culturable of A) *P. aeruginosa* and B) *A. baumannii* in CMPR bulk water. Error bars indicate mean ± standard deviation. Samples sizes are n=3 for CMPRs that received disinfectant, n=6 for disinfectant-free controls.

B)

A)

Figure S13**.** Differences in the relative abundance of A) *P. aerguginosa* and B) *A. baumannii* levels between disinfectant and disinfectant-free conditions in CMPR effluent. Symbols indicate statistical significance of difference via Dunn’s Test (● = p>0.05, **▲** = p<0.05). Mean remaining disinfectant residual following stagnation as a percentage of the NPDWS or MCL are displayed for each condition. Samples sizes are n=3 for CMPRs that received disinfectant, n=6 for controls.


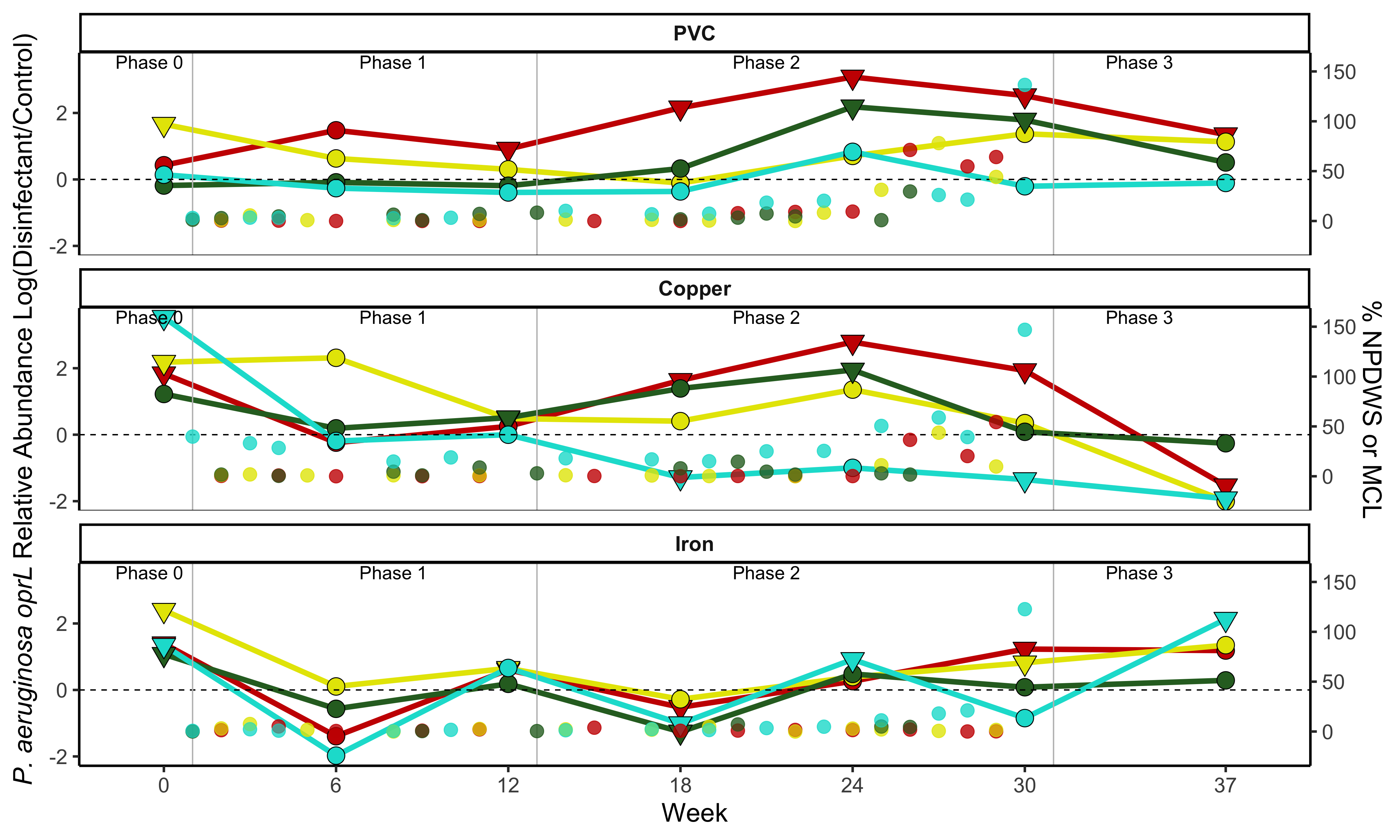

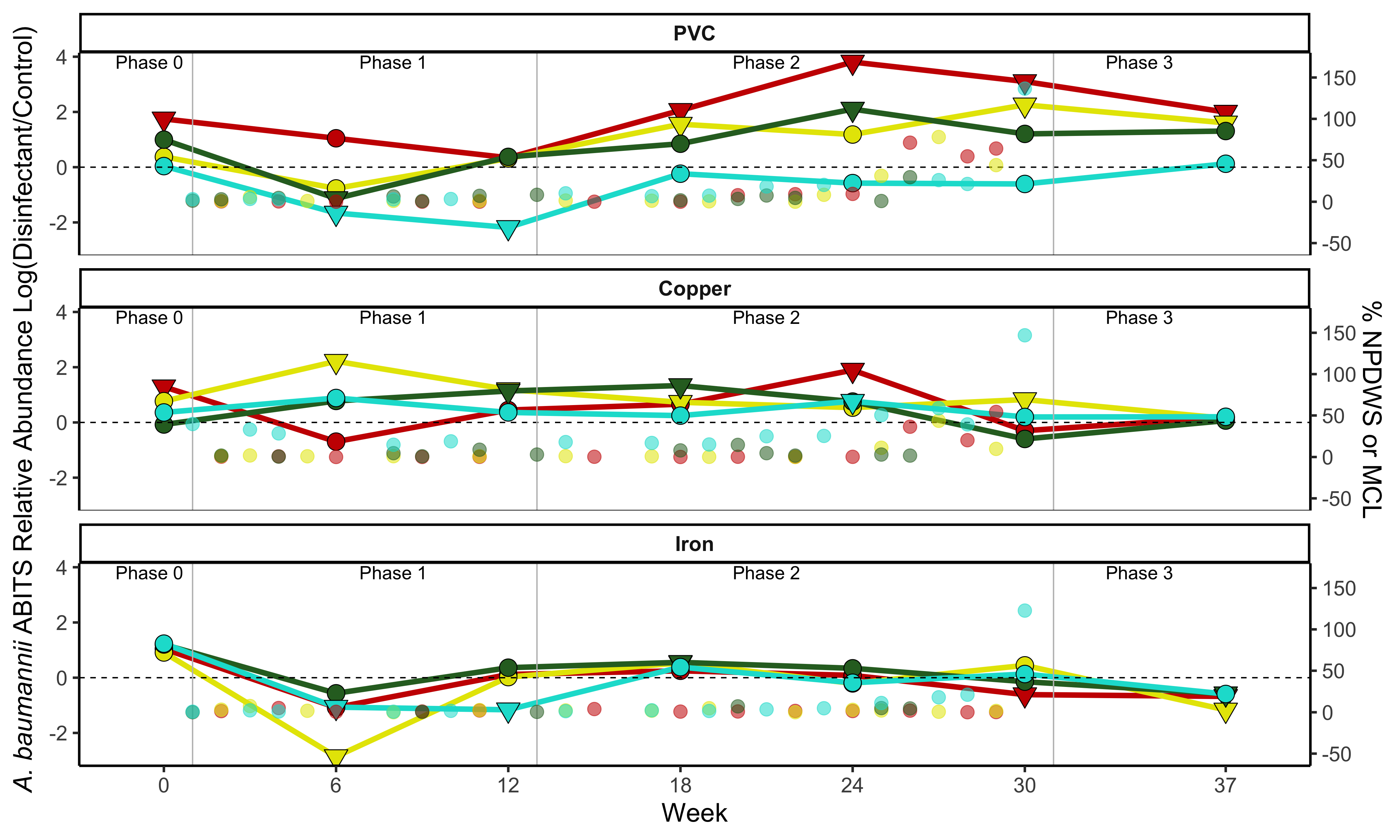


A)

B)


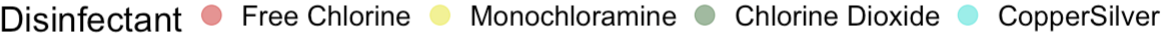


Measured disinfectant in effluent easdfeefsdal;fkjsadfeffleuntyeffluent


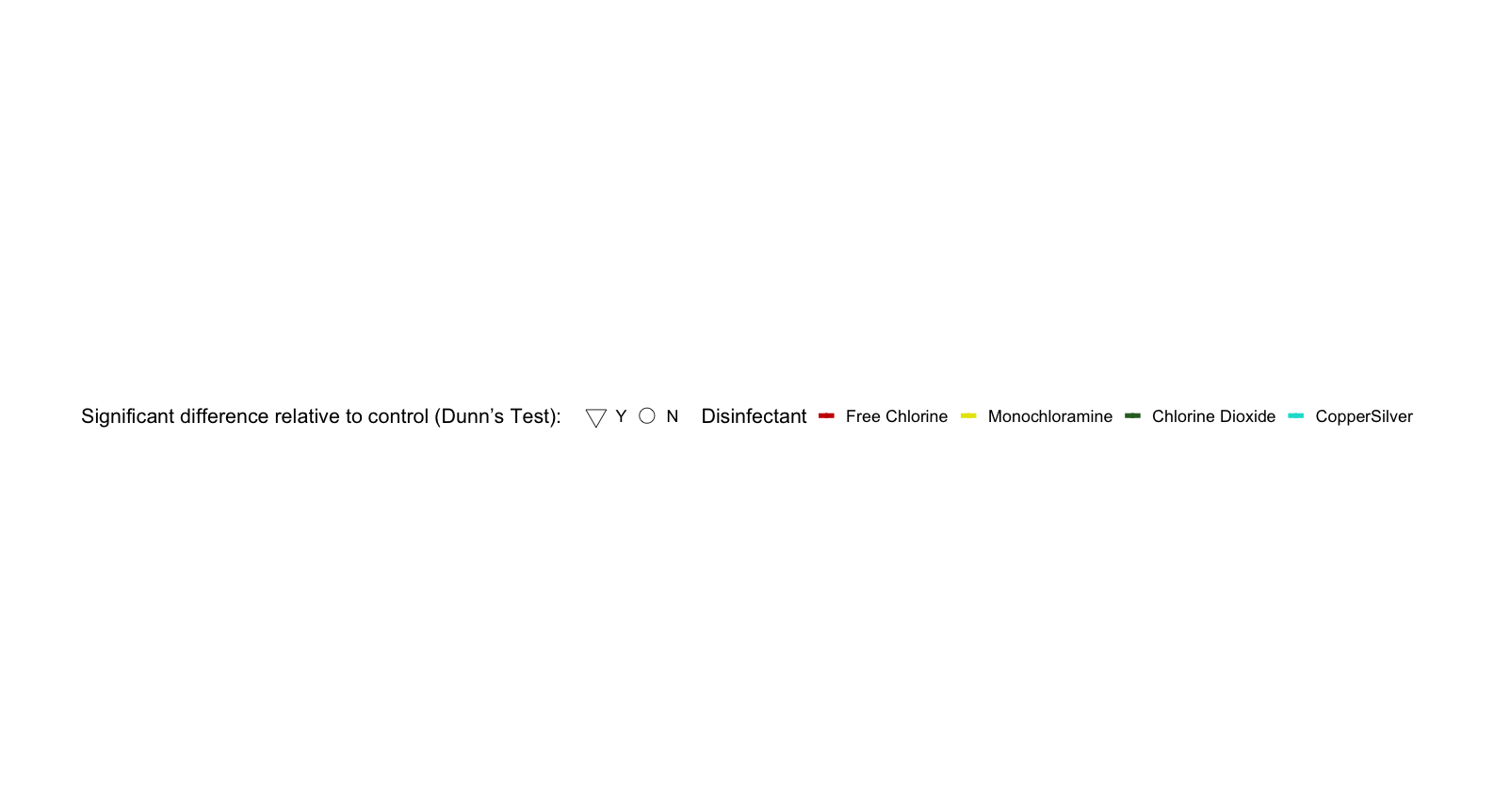

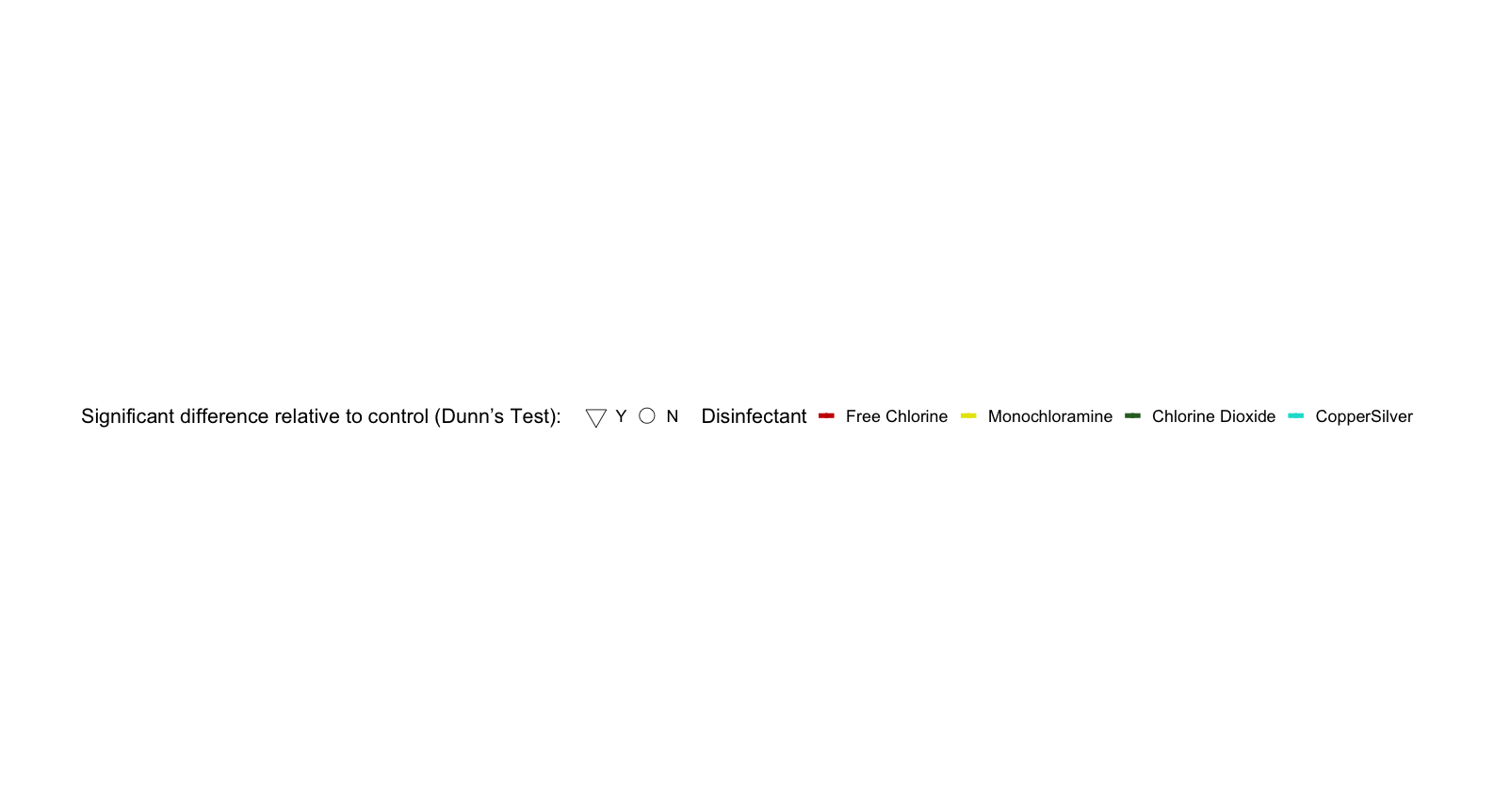


Disinfectant

Figure S14**.** Average relative abundance of A) *P. aeruginosa* and B) *A. baumannii* in CMPR bulk water. Error bars indicate mean ± standard deviation. Samples sizes are n=3 for CMPRs that received disinfectant, n=6 for disinfectant-free controls.
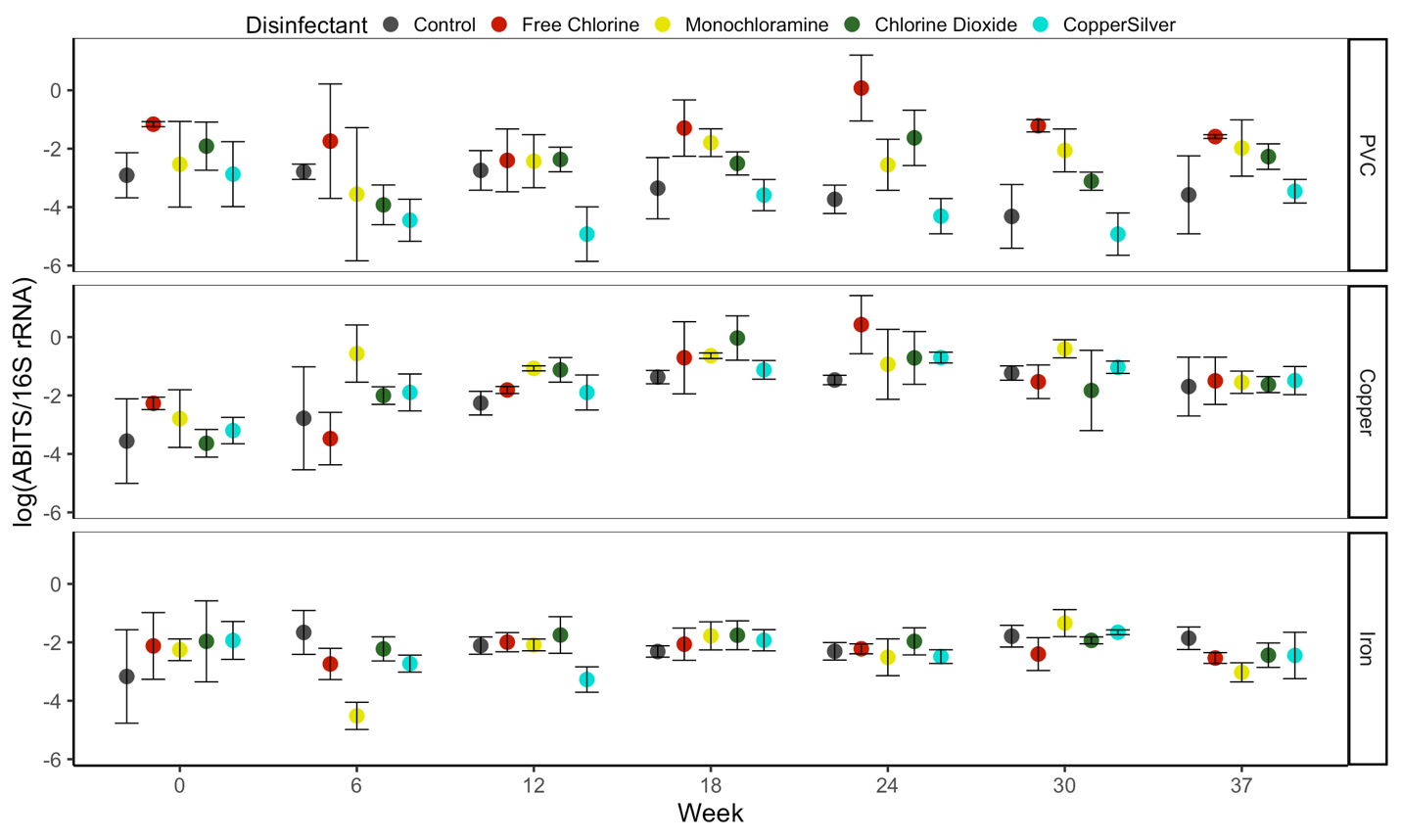

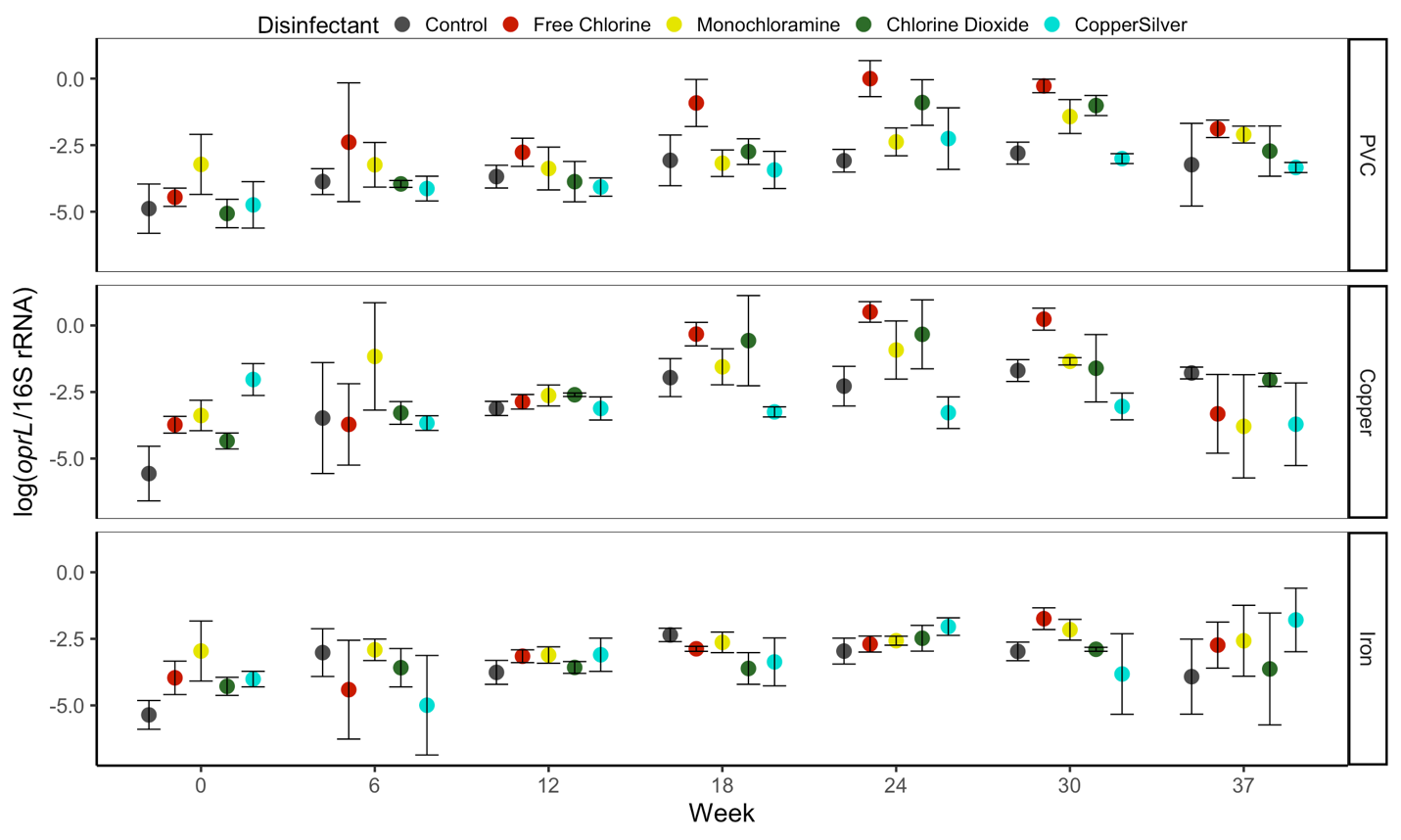


B)

A)


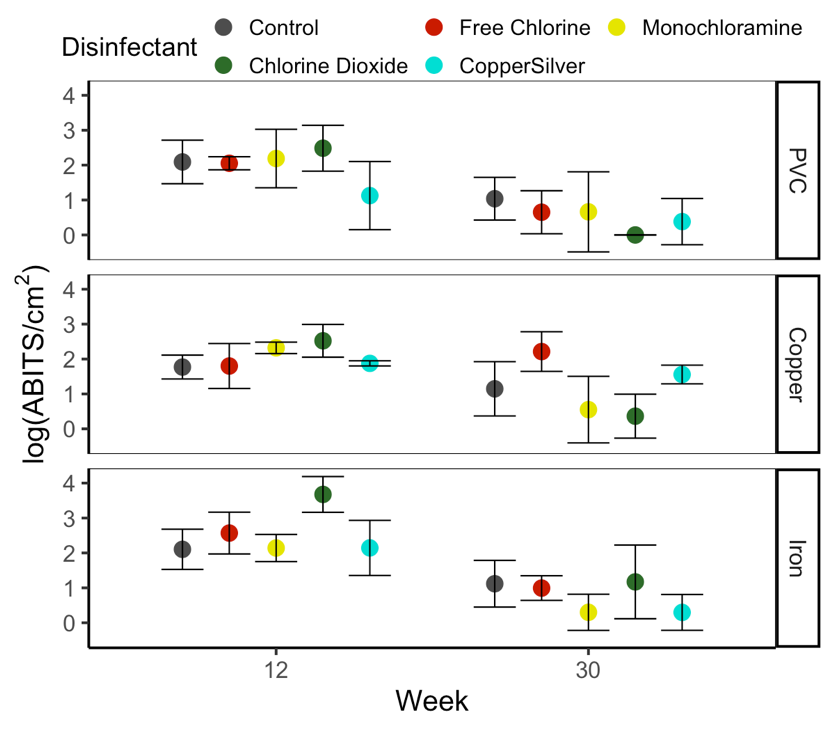

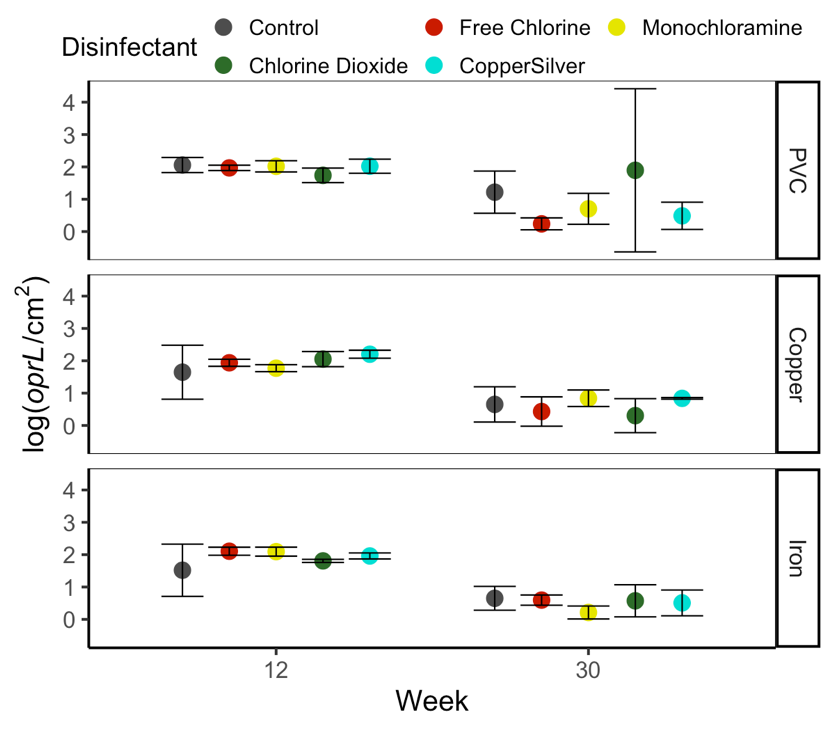

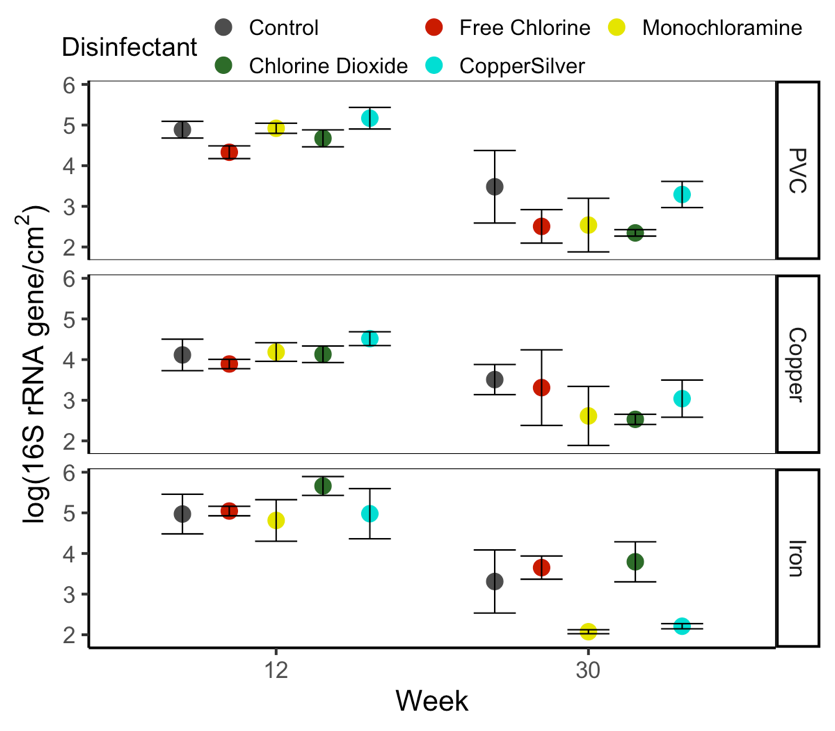


B)

A)

Figure S15**.** Average biofilm levels of A) total bacteria (16S rRNA gene copy numbers), B) *P. aeruginosa* (*oprL* gene copy numbers), and C) *A. baumannii* (16S-23S rRNA gene intergenic spacer (ABITS) copy numbers) per cm^2^. Error bars indicate mean ± standard deviation. Samples sizes are n=3 for CMPRs that received disinfectant, n=6 for disinfectant-free controls.

C)






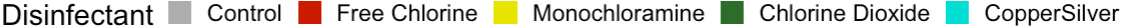


B)

A)

Figure S16**.** Differences in culturable A) *P. aeruginosa* and B) *A. baumannii* between disinfectant and disinfectant-free (control) conditions in the CMPRs at the end of Phase 2 (Week 30). Average log-transformed levels per cm^2^ in disinfectant-free conditions are displayed in gray, with error bars representing 95% non-parametric bootstrap confidence intervals. Asterisks (*) indicate statistically significant difference from disinfectant-free CMPRs via Dunn’s Test (p<0.05). Samples sizes are n=3 for CMPRs that received disinfectant, n=6 for controls.


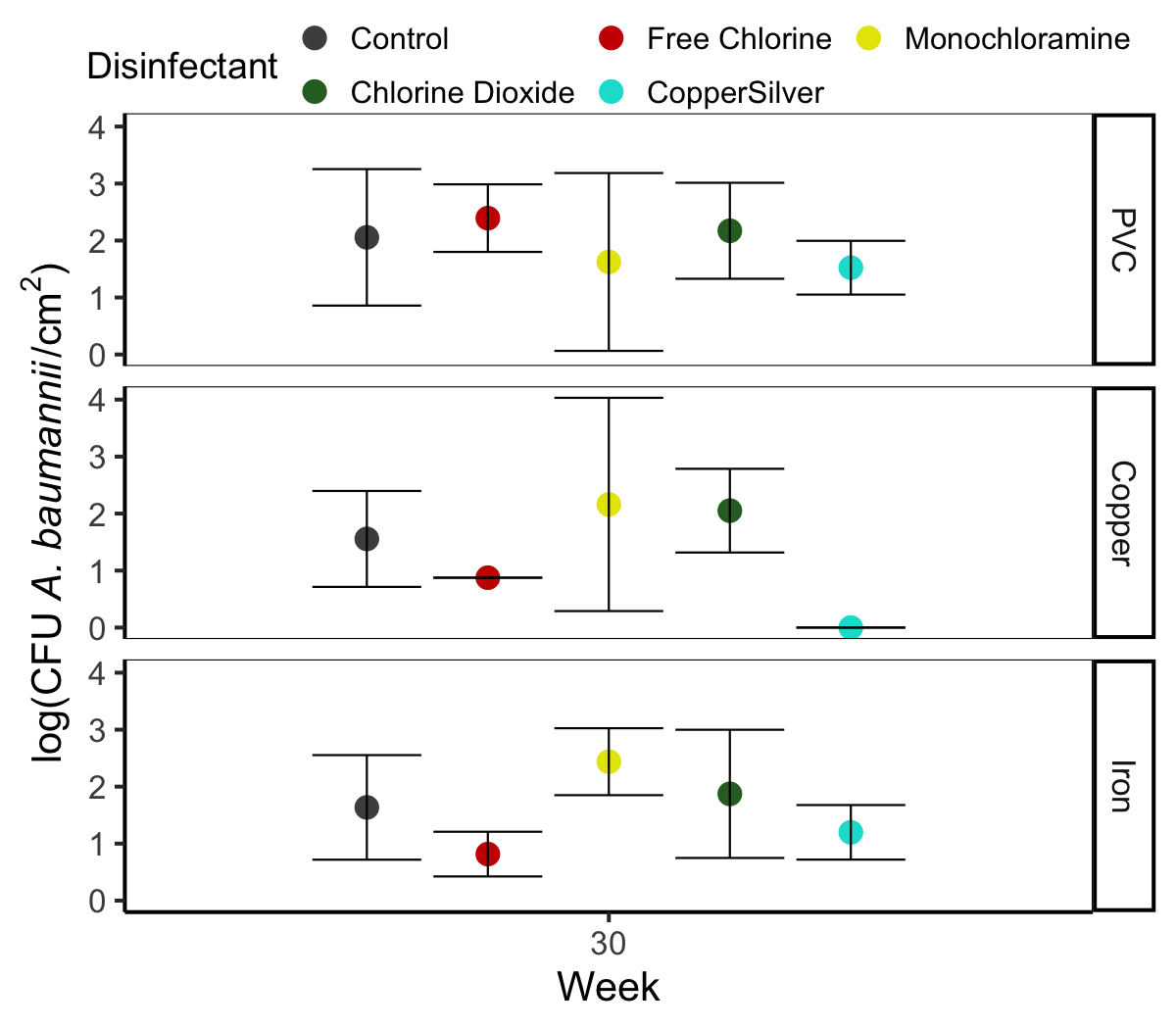

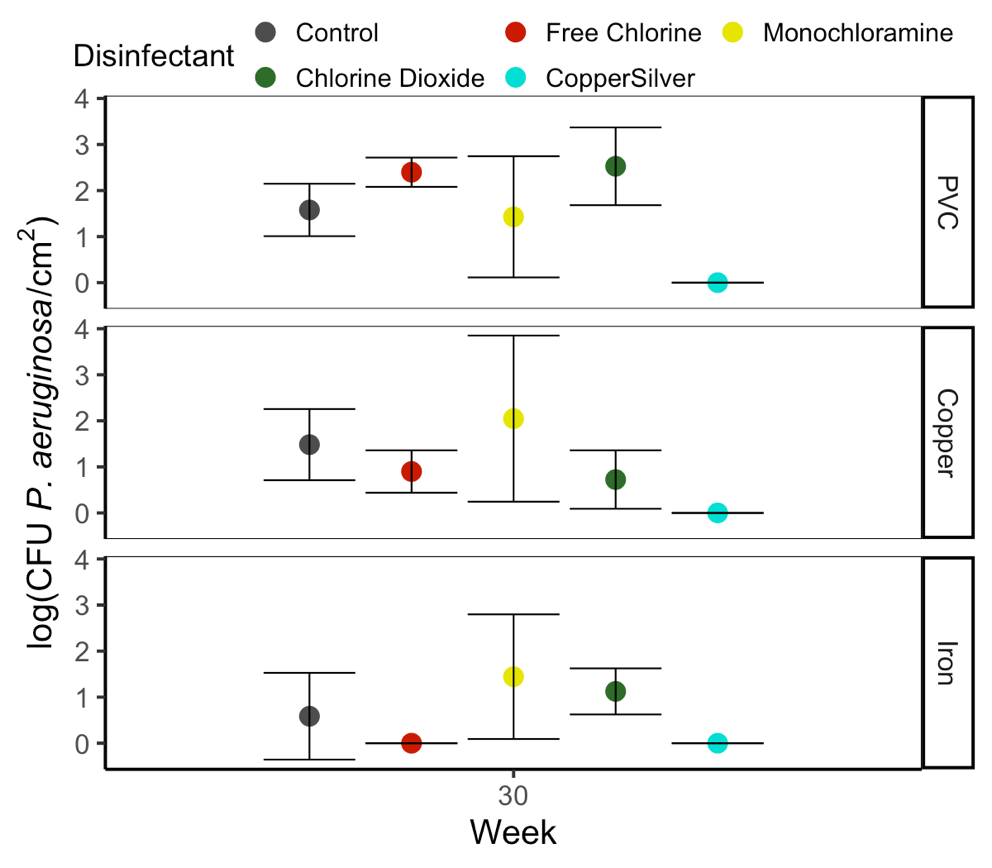
Figure S17**.** Average biofilm levels of culturable A) B) *P. aeruginosa* and B) per cm^2^ at the end of Phase 2 (Week 30). Error bars indicate mean ± standard deviation. Samples sizes are n=3 for CMPRs that received disinfectant, n=6 for disinfectant-free controls

Figure S18**.** Differences A) *P. aeruginosa* and B) *A. baumannii* as measured by culture and qPCR. Lines representing the linear regression between these two parameters are displayed for each disinfectant condition. n=42 for each pipe material for the disinfectant-free conditions and n=21 for disinfectant conditions.

Figure S19**.** Canonical correspondence analysis between biological parameters (red)— culturable *P. aeruginosa* (logCulturePA), *P. aeruginosa* gene copies (log_oprL), culturable *A. baumannii* (logCultuerAB), *A. baumannii* gene copies (log_ABITS), total bacteria (Log16S)—and disinfectant conditions and chemistry parameters (blue), including influent disinfectant dose and mean effluent disinfectant residual. Data were separated into A) PVC, B) copper-PVC, and C) iron-PVC CMPRs.

## References

1. Proctor, C.R.; Dai, D.; Edwards, M.A.; Pruden, A. Interactive effects of temperature, organic carbon, and pipe material on microbiota composition and Legionella pneumophila in hot water plumbing systems. *Microbiome* **2017**, *5*, 130, doi:10.1186/s40168-017-0348-5.

2. Suzuki, M.T.; Taylor, L.T.; DeLong, E.F. Quantitative Analysis of Small-Subunit rRNA Genes in Mixed Microbial Populations via 5′-Nuclease Assays. *Appl. Environ. Microbiol.* **2000**, *66*, 4605–4614, doi:10.1128/AEM.66.11.4605-4614.2000.

3. Le Gall, F.; Le Berre, R.; Rosec, S.; Hardy, J.; Gouriou, S.; Boisramé-Gastrin, S.; Vallet, S.; Rault, G.; Payan, C.; Héry-Arnaud, G. Proposal of a quantitative PCR-based protocol for an optimal Pseudomonas aeruginosa detection in patients with cystic fibrosis. *BMC Microbiol.* **2013**, *13*, 143, doi:10.1186/1471-2180-13-143.

4. Chang, H.C.; Wei, Y.F.; Dijkshoorn, L.; Vaneechoutte, M.; Tang, C.T.; Chang, T.C. Species-Level Identification of Isolates of the Acinetobacter calcoaceticus - Acinetobacter baumannii Complex by Sequence Analysis of the 16S-23S rRNA Gene Spacer Region. *J. Clin. Microbiol.* **2005**, *43*, 1632–1639, doi:10.1128/JCM.43.4.1632-1639.2005.

5. Wickham, H. tidyverse: Easily Install and Load the “Tidyverse.”; 2017.

6. Wickham, H.; Bryan, J. readxl: Read Excel Files 2019.

7. Dinno, A. dunn.test: Dunn’s Test of Multiple Comparisons Using Rank Sums 2017.

8. Mangiafico, S. rcompanion: Functions to Support Extension Education Program Evaluation 2020.

9. Auguie, B. Graphics, gridExtra: Miscellaneous Functions for “Grid” 2017.

10. Butterfield, C.T.; Wattie, E.; Megregian, S.; Chambers, C.W. Influence of pH and Temperature on the Survival of Coliforms and Enteric Pathogens When Exposed to Free Chlorine. *Public Heal. Reports* **1943**, *58*, 1837, doi:10.2307/4584715.

11. Song, Y.; Pruden, A.; Edwards, M.A.; Rhoads, W.J. Natural Organic Matter, Orthophosphate, pH, and Growth Phase Can Limit Copper Antimicrobial Efficacy for Legionella in Drinking Water. *Environ. Sci. Technol.* **2021**, *55*, 1759–1768, doi:10.1021/acs.est.0c06804.

12. Salehi, M.; Odimayomi, T.; Ra, K.; Ley, C.; Julien, R.; Nejadhashemi, A.P.; Hernandez-Suarez, J.S.; Mitchell, J.; Shah, A.D.; Whelton, A. An investigation of spatial and temporal drinking water quality variation in green residential plumbing. *Build. Environ.* **2020**, *169*, 106566, doi:10.1016/j.buildenv.2019.106566.

13. Spencer, M.S.; Cullom, A.C.; Rhoads, W.J.; Pruden, A.; Edwards, M.A. Replicable simulation of distal hot water premise plumbing using convectively-mixed pipe reactors. *PLoS One* **2020**, *15*, e0238385.

14. Stumm, W.; Morgan, J.J. *Aquatic chemistry: chemical equilibria and rates in natural waters*; John Wiley & Sons, 2012; Vol. 126; ISBN 1118591488.

15. Feely, R.A.; Alin, S.R.; Newton, J.; Sabine, C.L.; Warner, M.; Devol, A.; Krembs, C.; Maloy, C. The combined effects of ocean acidification, mixing, and respiration on pH and carbonate saturation in an urbanized estuary. *Estuar. Coast. Shelf Sci.* **2010**, *88*, 442–449, doi:10.1016/j.ecss.2010.05.004.

16. McNeill, L.S.; Edwards, M. Iron Pipe Corrosion in Distribution Systems. *J. Am. Water Works Assoc.* **2001**, *93*, 88–100, doi:10.1002/j.1551-8833.2001.tb09246.x.

17. Falkinham, J.; Falkinham III, J.O. Common Features of Opportunistic Premise Plumbing Pathogens. *Int. J. Environ. Res. Public Health* **2015**, *12*, 4533–4545, doi:10.3389/ fmicb.2014.00258.

18. Durand, M.L. Disinfectants and plumbing materials: Effects on the sensory and chemical characteristics of drinking water, Virginia Tech: Blacksburg, VA, USA, 2005.

19. Eisnor, J.D.; Gagnon, G.A. Impact of secondary disinfection on corrosion in a model water distribution system. *J. Water Supply Res. Technol.* **2004**, *53*, 441–452, doi:10.2166/aqua.2004.0035.

20. Eaton, A.D.; Clesceri, L.S.; Greenberg, A.E.; Franson, M.A.H. Standard methods for the examination of water and wastewater. In; American Public Health Association, American Water Works Association, Water Environment Federation: Washington, DC, USA, 1995.

21. Zhang, H.; Tian, Y.; Kang, M.; Chen, C.; Song, Y.; Li, H. Effects of chlorination/chlorine dioxide disinfection on biofilm bacterial community and corrosion process in a reclaimed water distribution system. *Chemosphere* **2019**, *215*, 62–73, doi:10.1016/j.chemosphere.2018.09.181.

22. Bédard, E.; Charron, D.; Lalancette, C.; Déziel, E.; Prévost, M. Recovery of Pseudomonas aeruginosa culturability following copper- and chlorine-induced stress. *FEMS Microbiol. Lett.* **2014**, *356*, 226–234, doi:10.1111/1574-6968.12494.

23. Dopp, E.; Richard, J.; Dwidjosiswojo, Z.; Simon, A.; Wingender, J. Influence of the copper-induced viable but non-culturable state on the toxicity of Pseudomonas aeruginosa towards human bronchial epithelial cells in vitro. *Int. J. Hyg. Environ. Health* **2017**, *220*, 1363–1369, doi:10.1016/j.ijheh.2017.09.007.

24. Grobe, S.; Wingender, J.; Flemming, H.-C. Capability of mucoid Pseudomonas aeruginosa to survive in chlorinated water. *Int. J. Hyg. Environ. Health* **2001**, *204*, 139–142, doi:10.1078/1438-4639-00085.

25. Bédard, E.; Prévost, M.; Déziel, E. Pseudomonas aeruginosa in premise plumbing of large buildings. *Microbiologyopen* **2016**, *5*, 937–956, doi:10.1002/mbo3.391.

26. Wang, Y.; Zhang, X.; Feng, S.; Niu, Z.; Chen, C. Study on inactivation of iron bacteria isolated from real drinking water distribution systems by free chlorine and chloramine. *Ann. Microbiol.* **2009**, *59*, 353–358, doi:10.1007/BF03178339.
